# Mitochondrial unfolded protein response gene CLPP changes mitochondrial dynamics and affects mitochondrial function

**DOI:** 10.7717/peerj.7209

**Published:** 2019-07-02

**Authors:** GuiJun Wu, Qing Xiong, XiaoJun Wei, Ye Wang, XueMei Hu, GuangZhen He, LinJie Liu, QianHui Lai, Zhe Dai, Dhakal Anushesh, Yancheng Xu

**Affiliations:** 1 Department of Endocrinology, Zhongnan Hospital of Wuhan University, Wuhan, China; 2 Emergency Centre, Zhongnan Hospital of Wuhan University, Wuhan, China

**Keywords:** Caseinolytic peptidase P, Mitochondrial unfolded protein response, Mitochondrial dynamics, Fusion, Fission

## Abstract

Mitochondrial dynamics is associated with mitochondrial function, which is associated with diabetes. Although an important indicator of the mitochondrial unfolded protein response, to the best of our knowledge, *CLPP* and its effects on mitochondrial dynamics in islet cells have not been studied to date. We analyzed the effects of *CLPP* on mitochondrial dynamics and mitochondrial function in the mice islet β-cell line Min6 under high glucose and high fat conditions. Min6 cells were assigned to: Normal, HG, HG+NC, HG+si*CLPP*, HF, HF+NC and HF+ si*CLPP* groups. High glucose and high fat can promote the mRNA and protein expression of *CLPP* in mitochondria. The increase of mitochondrial fission, the decrese of mitochondrial fusion, and the damage of mintocondrial ultrastructure were significant in the si*CLPP* cell groups as compared to no-si*CLPP* treated groups. Meanwhile, mitochondrial functions of MIN6 cells treated with si*CLPP* were impaired, such as ATP decreased, ROS increased, mitochondrial membrane potential decreased. In addition, cell insulin secretion decreased and cell apoptosis rate increased in si*CLPP* groups. These results revealed that mitochondrial unfolded protein response gene*CLPP* alleviated high glucose and high fat-induced mitochondrial dynamics imbalance and mitochondrial dysfunction.

## Introduction

Type 2 diabetes mellitus (T2DM) is a growing, worldwide epidemic characterized as abnormal insulin secretion by pancreatic β-cells and peripheral insulin resistance, resulting in high blood glucose. As mitochondria is involved in multiaspect cellular roles (production of energy and regulation of several signaling cascades including the redox state, calcium signaling, substrate metabolism, cell survival, and apoptotic pathways) (Alston et al., 2017; Finkel, 2012; Quiros, Mottis & Auwerx, 2016; Rizzuto et al., 2012; Wang & Youle, 2009), mitochondrial dysfunction is intimately linked to the pathogenesis of pancreatic β-cell damage in T2DM. Accumulating data indicate that mitochondrial dynamic is essential to mitochondrial function. Mitochondria constitutes a dynamic network that undergoes frequent mitochondrial fusion and fission for regulating and maintaining mitochondrial shape, structure, quantity, and function (Mishra & Chan, 2016). Therefore, mitochondria is not a static organelle. Mitochondria exists in a state of dynamic balance between fusion and fission in physiological conditions, and this homeostasis can be changed when the supply and demand of energy is changed, leading to mitochondrial dysfunction and even mitophagy (Green & Kroemer, 2004). Several dynamin-related GTPases constitute the main steps of mitochondrial fusion-fission process. Proteins responsible for fusion are mitofusin-1 (*MFN1*), mitofusin-2 (*MFN2*), and optic atrophy (*OPA1*). Proteins responsible for fission are dynamin-related protein 1 (*DRP1*) and mitochondrial fission 1 (*FIS1*) (Chan, 2012; Chen et al., 2003; Cipolat et al., 2004; Ishihara et al., 2006; Mishra & Chan, 2016; Song et al., 2007). Knockout out of *OPA1* results in fragmentation of the mitochondrial endoplasmic reticulum, suggesting that the disruption of the mitochondrial organelle can affect mitochondrial morphology (Griparic, Kanazawa & Van der Bliek, 2007; Kushnareva et al., 2013). Similarly, cell lacking *MFN1* and *MFN2* also showed mitochondrial fragmentation. In *MFN2* deficiency, the fusion organelle formed by *MFN1* and *OPA1* is still available. This suggests that *MFN1* and *OPA1* are essential for the mitochondrial fusion, while *MFN2* is dispensable (Cipolat et al., 2004; Zanna et al., 2008). The regulation of mitochondrial fusion and fission is a complex process involving different proteins that alter expression in response to stress and signals.

Caseinolytic peptidase P (*CLPP*) is a member of the *CLP* family (caseinolytic protease, *HSP100*). It is a highly conserved ATP-dependent protease and is widely distributed from prokaryotic cells to eukaryotic organelles (Baker & Sauer, 2012; Yu & Houry, 2007). *CLPP* has been extensively studied in prokaryotes, whereas the role of *CLPP* in mammalian mitochondrial is far less known (Corydon et al., 1998; Rath et al., 2012). *CLPP* is found to participate in the progress of mitochondrial unfolding protein response (mtUPR) (Al-Furoukh et al., 2015; Voos et al., 2016). In the human embryonic kidney cell line, HEK293T, and mice myoblasts, C2C12, the overexpression of *CLPP* is correlated with the expression of genes involved in the mtUPR (Al-Furoukh et al., 2015). However, there has been recent research that questions this role of *CLPP* (Seiferling et al., 2016). Unfolded or misfolded proteins accumulate in the mitochondrial matrix in response to environmental insults (Powers & Balch, 2013; Rath et al., 2012). The mtUPR can hydrolyze protein and reduce the amount of unfolded proteins in the mitochondria to maintain proteostasis and mitochondrial function (Mouchiroud et al., 2013; Quiros, Langer & Lopez-Otin, 2015).

At present, the relationship between *CLPP* and mitochondrial dynamics under high glucose and high fat conditions in islet endocrine cells has not been reported. In this study, we aimed to uncover the role of *CLPP* in mitochondrial dynamics and mitochondrial function in the presence of high glucose and high fat.

## Materials and Methods

### Cell culturezz

Pancreatic Min6 beta cells were purchased from the Cell Bank of the Chinese Academy of Sciences. Min6 cells were routinely cultured in RPMI-1640 (SH30809.01; GE Healthcare, Salt Lake City, UH, USA) supplemented with 20% fetal bovine serum (FBS), 1% glutamine, 1% β-mercaptoethanol, and 10 mM HEPES. Cells were cultured in a 37 °C with 5% CO_2_ incubator. The medium was refreshed every 24 h. Palmitic acid (P0500, PA; Sigma-Aldrich, Carlsbad, CA, USA) was conjugated with fatty-acid-free bovine serum albumin (BSA) (A8020; Solarbio, Beijing, China) before addition to cell culture. PA was dissolved in 99% ethanol and then mixed with 10% BSA in serum-free DMEM (SH30022.01; GE Healthcare, Salt Lake City, UT, USA) to make a 50 mM PA stock solution. Different concentrations of glucose and palmitic acid were prepared in RPMI-1640 medium. The osmotic pressure was adjusted with D-mannitol (0122; Amresco, Seattle, Washington, USA). Min6 cells were treated with glucose (5.6,16.7 , and 33.3 mmol/l) and palmitic acid (0.2, 0.5, and 1.0 mmol/l) for 24 h, 36 h, and 48 h.

### Cell grouping

Part 1: before cell transfection, Min6 Cells were assigned to the normal group (routine culture), the groups with different concentrations of glucose (5.6 mmol/l, 16.7 mmol/l and 33.3 mmol/l) and the groups with different concentrations of palmitic acid (0.2 mmol/l, 0.5 mmol/l and 1.0 mmol/l). Part 2: after cell transfection, Min6 Cells were assigned to the normal group (routine culture), the HG group (treated with 33.3 mmol/l glucose), the HG + NC group (transfected with the *CLPP* negative sequence and then treated with 33.3 mmol/l glucose), the HG + si*CLPP* group (transfected with *CLPP* inhibitor sequence and then treated with 33.3 mmol/l glucose ), the HF group (treated with 0.5 mmol/l fat), the HF +NC group (transfected with the *CLPP* negative sequence and then treated with 0.5 mmol/l fat), and the HF + *siCLPP* group (transfected with the CLPP inhibitor sequence and then treated with 0.5 mmol/l fat).

### Real-time quantitative PCR (qPCR)

Total RNA was extracted by using the RNAprep Pure Cell/Bacteria Kit (Code No. DP430, Qiagen, Beijing, China). 2 µg RNA was reversed transcribed into cDNA by using the PrimeScript™ RT Reagent Kit (Perfect Real Time) (Code No. RR037A; TaKaRa, Dalian, China). qPCR was performed using TB Green™ Premix Ex Taq™ II (Tli RNaseH Plus) (Code No. RR820A; TaKaRa, Dalian, China). qPCR was carried out as follows: initial denaturation at 95 °C for 30 s; 40 thermal cycles at 95 °C for 5 s; and 60 °C for 30 s. A CFX96™ real-time system (Bio-Rad, Hercules, CA, USA) was used for qRT-PCR analysis. β-actin was used as an internal control. Each group was detected in triplicate, and data were calculated according to the 2^−ΔΔ*Ct*^ method and normalized to the control. The primer sequences of genes were shown in Table 1.

**Table 1:** Primers used in the study (mouse).

GenBank accession number	Primer	Sequence	Length (bp)
NM_017393	Clpp (F)	5′-GCCTTGCCGTGCATTTCTC-3′	113
	Clpp (R)	5′-CTCCACCACTATGGGGATGA-3′	
NM_024200	Mfn1 (F)	5′-CCTACTGCTCCTTCTAACCCA-3′	86
	Mfn1 (R)	5′-AGGGACGCCAATCCTGTGA-3′	
NM_133201	Mfn2 (F)	5′-AGAACTGGACCCGGTTACCA-3′	82
	Mfn2 (R)	5′-CACTTCGCTGATACCCCTGA-3′	
NM_001199177	Opa1 (F)	5′-TGGAAAATGGTTCGAGAGTCAG-3′	77
	Opa1 (R)	5′-CATTCCGTCTCTAGGTTAAAGCG-3′	
NM_001025947	Drp1 (F)	5′-TTACGGTTCCCTAAACTTCACG-3′	74
	Drp1 (R)	5′-GTCACGGGCAACCTTTTACGA-3′	
NM_025562	Fis1 (F)	5′-AGAGCACGCAATTTGAATATGCC-3′	125
	Fis1 (R)	5′-ATAGTCCCGCTGTTCCTCTTT-3′	
NM_026880	Pink1 (F)	5′-TTCTTCCGCCAGTCGGTAG-3′	141
	Pink1 (R)	5′-CTGCTTCTCCTCGATCAGCC-3′	
NM_016694	Parkin (F)	5′-TCTTCCAGTGTAACCACCGTC-3′	115
	Parkin (R)	5′-GGCAGGGAGTAGCCAAGTT-3′	
NM_007393	Actin (F)	5′-GGCTGTATTCCCCTCCATCG-3′	154
	Actin (R)	5′-CCAGTTGGTAACAATGCCATGT-3′	

### Western blotting

Protein was extracted by using RIPA (G2002; Servicebio, Wuhan, China) with 1 mmol/l phenylmethanesulfonyl fluoride (PMSF) (ST505; Beyotime Biotechnology, Shanghai, China) at 4 °C for 30 min and centrifuged at 12,000 rpm for 10 min. The extracted protein was boiled for 15 min. The protein concentration was detected by using an enhanced BCA protein assay kit (Code No. P0010; Beyotime Biotechnology, Shanghai, China). 40 µg protein was separated by 10% sodium dodecyl sulfate polyacrylamide gel electrophoresis (SDS-PAGE) and transferred to polyvinylidene fluoride (PVDF) membranes (IPVH00010, Millipore, USA). The membrane was incubated with 5% defatted milk at 4 °C overnight, and incubated with primary antibodies against *CLPP* (66271-1-Ig, 1:1000; Proteintech, USA), *MFN1* (ab234861, 1:1000; Abcam, USA), *MFN2* (CB1020, 1:500; Sigma, Kanagawa, Japan), *OPA1* (27733-1-AP, 1:500; Proteintech, USA), *DRP1* (12957-1-AP, 1:500, Proteintech, Chicago, IL, USA), *FIS1* (ab71498, 1:1000, Abcam, Burlingame, CA, USA) or β*-actin* (GB12001, 1:3000; Servicebio, Wuhan, China) for 2 h, and then incubated with secondary antibody respectively (GB23303, 1:3000; Servicebio, Wuhan, China) at room temperature for 30 min. The blots were detected using Bio-Rad Molecular Imager ChemiDoc XRS+ (USA).

### Cell transfection

The siRNA duplexes used in this study were purchased from Invitrogen (NY, USA) and had the following sequence: 5′-CAGGUGAUCGAGUCAGCAAUGUUGCUGACUCGAUCACCUGUA-3′. Negative Control (Invitrogen, NY, USA) was used as the control. Min6 cells were seeded on 6-well plates. When the cell density reached 70–80%, cells were transfected using Lipofectamine 2000 kit (11668019; Invitrogen, Carlsbad, CA, USA). After 48 h, the culture medium was replaced by complete culture medium to stop transfection. Cells were harvested for the next experiment.

### ATP assays

Intracellular ATP content was measured using an enhanced ATP assay kit (Code No. S0026; Beyotime Biotechnology, Shanghai, China) according to the manufacturer’s instructions. In briefly, cells were lysed using ATP lysate buffer and then centrifuged at 12,000× g for 10 min at 4 °C. The supernatant was removed and mixed with dilution buffer containing luciferase. The relative light units were measured by a microplate luminometer according to the manufacturer’s instructions. A fresh standard curve was prepared each time and ATP content was calculated using the curve.

### Insulin secretion assay

Secreted insulin was measured using the Mouse Insulin (INS) ELISA Kit (Code No. CSB-E05071 m; Cusabio, Wuhan, China). The Assay Layout Sheet was used as a reference to determine the number of wells to be used. 100 µl of standard or sample per well was then added. After incubation for 2 h at 37 °C, the liquid was removed from each well. 100 µl of Biotin-antibody (1 ×) was added to each well, followed by incubation for 1 h at 37 °C. Each well was aspirated and washed for three times. Then, 100 µl of HRP-avidin (1 ×) was added and incubated for 1 h at 37 °C. 90 µl of TMB substrate was then added and incubation for a further 15–30 min at 37 ° C. 50 µl of stop solution was added to each well. Optical density was determined within 5 min using a microplate reader set to 450 nm.

### Measurement of reactive oxygen species (ROS) generation

Cellular ROS contents were measured using the Reactive Oxygen Species Assay Kit (S0033; Beyotime Biotechnology, Shanghai, China) by flow cytometry (Beckman, Brea, CA, USA).Briefly, cell suspensions were transferred into centrifugal tubes with 1,500 rpm centrifugation for 5 min to collect cells. Cells were re-suspended with PBS (pre-cooled at 4 °C) and centrifuged at 1,500 rpm for 5 min. The number of cells was adjusted to 1 ×10^6^–2 ×10^8^ /ml. The probe DCFH-DA (10 µmol/l) was incubated with cell at 37 °C for 20 min to make full contaction. The cells were washed with serum-free medium for three times with to remove the probes that did not enter the cells. Flow cytometry was used to detect the ROS levels with an excitation wavelength of 488 nm and emission wavelength of 525 ± 20 nm. FlowJo software was used to analyze the ROS levels of cells.

### Mitochondrial membrane potential measurement

We detected the mitochondrial membrane potential (ΔΨ) with a fluorescent probe, JC-1 (5,5′,6,6′-tetrachloro-1,1′,3,3′-tetraethyl benzimidazolo-carbocyanine iodide). 1 ml JC-1 staining solution (mitochondrial membrane potential assay kit with JC-1; C2006, Beyotime, Shanghai, China) was added to the cell and. incubated for 20 min at 37 °C. Cells were washed twice with JC-1 staining buffer (1X). When JC-1 monomer was detected, the excitation wavelength was set to 490 nm and the emission wavelength was set to 530 nm. When JC-1 polymer was detected, the excitation wavelength was set to 525 nm and the emission wavelength was set to 590 nm. JC-1 was aggregated in the mitochondrial matrix to form a polymer, which produced red fluorescence. JC-1 could not be aggregated in mitochondrial matrix to form a monomer, which produced green fluorescence. The appearance of red fluorescence indicated that mitochondrial membrane potential was normal and the mitochondrial state was normal. Green fluorescence showed that mitochondrial membrane potential was decreased and the mitochondrial state was abnormal. The results were observed under a fluorescence microscope. The ratio of red/green fluorescent densities was calculated with the Image Pro Plus 6.0 software.

### Mito-Tracker Red CMXRos Staining

Cells were stained with Mito-Tracker Red CMXRos dye (C1049; Beyotime, Shanghai, China) and DAPI (4′, 6-diamidino-2-phenylindole) (C1002; Beyotime, Shanghai, China). In briefly, when the cell density reached 70–80% in the cell culture plate, the cell culture medium was removed. Mito-Tracker Red CMXRos was added and the cells were incubated for 30 min at 37 °C. Then, Mito-Tracker Red CMXRos was removed and fresh cell culture medium was added. After dyeing, 4% polyformaldehyde (P0099) was added to fix cells at room temperature for 30 min. After fixing, cells were washed 3 times with Hank’s Balanced Salt Solution. The cells were incubated for 5 min at room temperature in DAPI labeling solution. The cells were washed three times in PBS. Nikon fluorescence microscopy (Tokyo, Japan) was used for observation. Integral optical density (IOD) was used for quantitative analysis of red light intensity. Image Pro Plus 6.0 software was used to analyze IOD values.

### Flow cytometry detection of cell apoptosis

Cells apoptosis were analyzed using the Annexin V-FITC Apoptosis Detection Kit; C1062S; Beyotime Biotechnology, Shanghai, China by a Beckman CytoFLEX FCM Flow Cytometer (Brea, CA, USA). The green fluorescence of Annexin V-FITC was detected using the FITC channel (FL1), and the red fluorescence of PI was detected using the PI channel (FL2). The parameters of flow cytometry were as follows: excitation wavelength Ex = 488 nm, emission wavelength FL1 (Em = 525 ± 20 nm) and FL2 (Em = 585 ± 21 nm). FlowJo software was used to analyze the proportion and number of different cell states (mainly early apoptosis, late apoptosis, and death).

### Transmission electron microscopy (TEM)

TEM was used to observe mitochondria ultra-structural of cells. Cells were fixed with 2.5% glutaraldehyde in a 0.1 M sodium phosphate buffer (pH 7.4, 4 °C, 2 h), post fixed in 2% osmium tetroxide in a 0.1 M phosphate buffer (4 °C, 1.5 h) and dehydrated in a graded series of concentrations of ethanol (30%, 50%, 70%, 90%, 96%, and 4 × 100%, each for 15 min), acetone (2 × 15 min) and embedded in epoxy resin (Epoxy Embedding Medium Kit; Sigma, Carlsbad, CA, USA). Ultra-thin sections (70 nm) were cut on a Leica Ultracut UCT25 ultramicrotome (German). After staining with uranyl acetate and lead citrate (each for 20 min), sections were examined using a TEM (FEI Tecnai G2 20 TWIN; Bionand). The area of mitochondria and the total cell were circled and calculated by AOI tool of Image Pro Plus 6.0 software.

### Statistical analysis

Data were expressed as the mean and standard deviation. Student *t*-tests or one-way analysis of variance (SPSS 18.0) were used to analyze. *P* < 0.05 was considered to be significant.

## Results

### High glucose and high fat increased the expression of *CLPP* in Min6 cells

**Figure 1:**
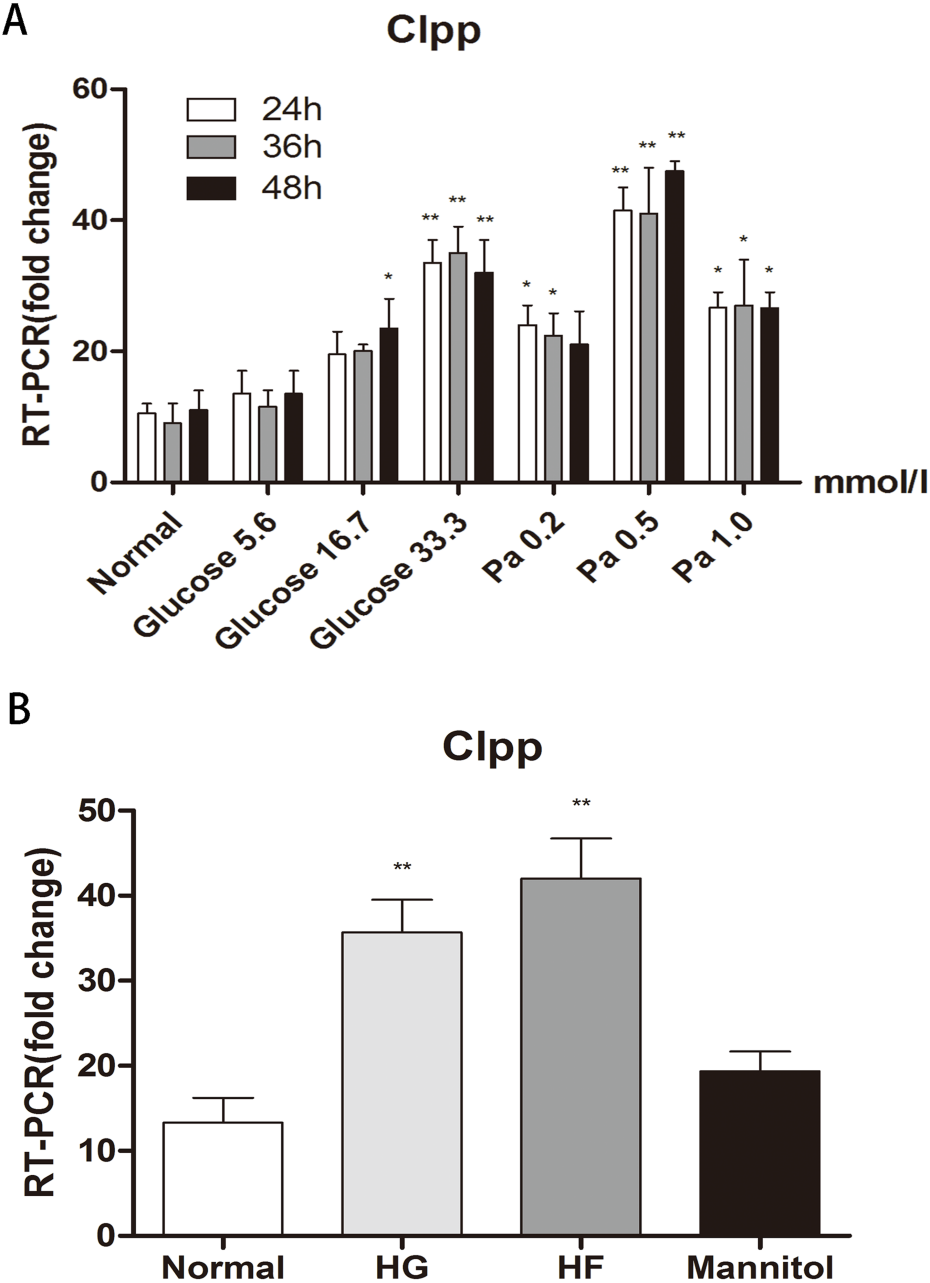
Effects of glucose and fat on the expression of CLPP. (A) RT-PCR detection of CLPP expression at 24 h, 36 h, 48 h after different concentrations of glucose and palmitic acid. (B) RT-PCR results of high glucose (33.3 mmol/l) and high fat (0.5 mmol/l) treatment for 48 h. *p < 0.05, **p < 0.01 versus normal.

Different concentrations of glucose and palmitic acid for different durations of treatment were adopted to detect the expression of *CLPP* in Min6 cells. After 24 h, 36 h and 48 h of treatment, in groups with different concentrations of glucose (5.6 mmol/l, 16.7 mmol/l and 33.3 mmol/l) and different concentrations of palmitic acid (0.2 mmol/l, 0.5 mmol/l and 1.0 mmol/l), the highest expression of *CLPP* was found in the groups with 33.3 mmol/l glucose or 0.5 mmol/l palmitic acid for 48 h compared with the normal group (*p* < 0.01) (Fig. 1A) (Raw data is available as Dataset 1). There were time-dependent and dose-dependent effects of glucose and fat on the expression of *CLPP,* but not in the 1 mmol/l palmitic acid group*. CLPP* expression was significantly increased at 48 h in the groups with high glucose and high fat compared with the normal group; this was evidenced by a 3.15-fold increase in the HG group and a 4.39-fold increase in the HF group (*p* < 0.01) (Fig. 1B) (Raw data is available as Dataset 2). Mannitol was used as a control to prove that osmotic pressure had no effect on the experiment. The results showed that high glucose and high fat could increase the expression of *CLPP*.

### High glucose and high fat inhibited mitochondrial fusion and promoted mitochondrial fission in Min6 cells

**Figure 2:**
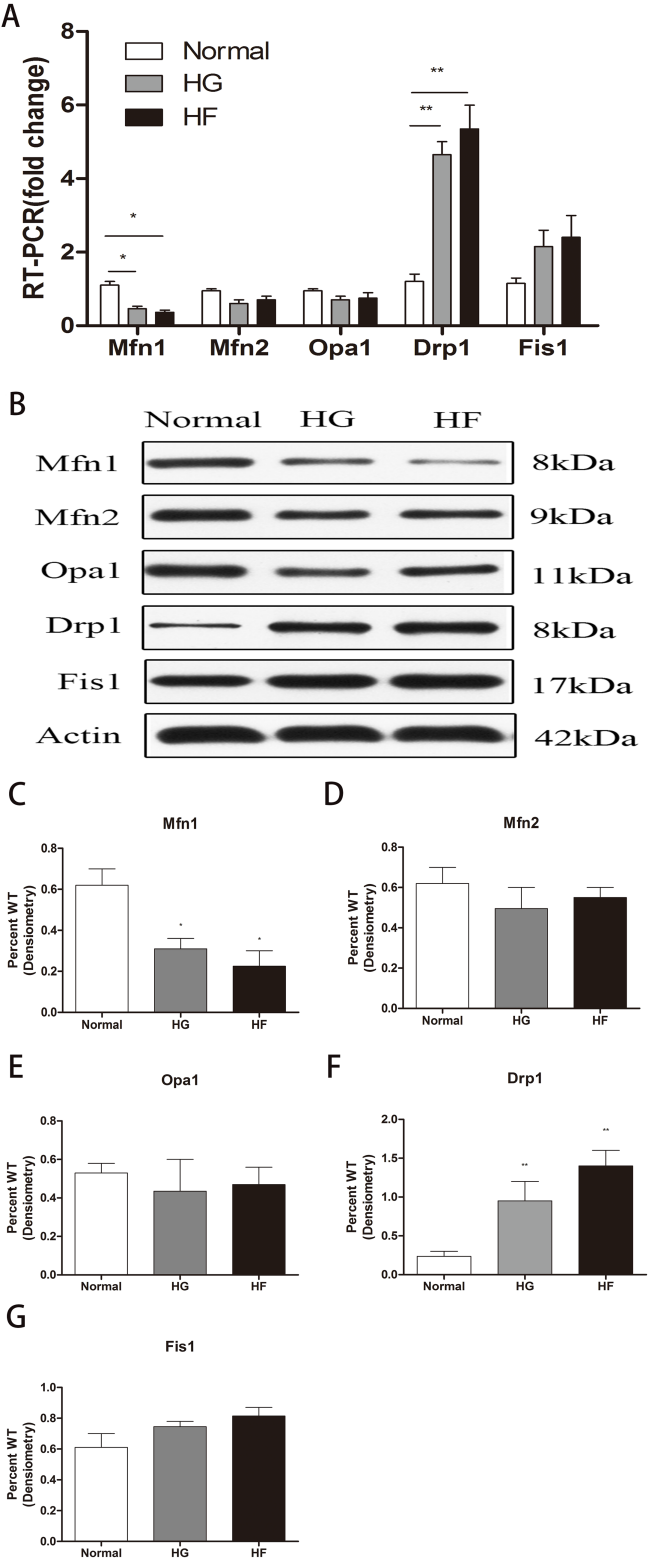
Effects of high glucose and high fat on mitochondrial dynamics-related proteins. (A) RT-qPCR showed the expression levels of mitochondrial dynamics-related proteins. (B) Western blot showed the protein levels of mitochondrial dynamics. (C–G) Statistical analysis of the expression of mitochondrial dynamics-related proteins detected by western blot in cells as described in B. *p < 0.05, **p < 0.01 versus normal.

The expression of related proteins in mitochondrial fusion and fission after high glucose and high palmitic acid treatment was detected. *MFN1* mRNA levels were expressed at lower levels in Min6 cells relative to the normal group in the HG and HF groups (reduced to 47% and 36%, respectively) (*p* < 0.05) (Fig. 2A) (raw data is available as Dataset 3). In addition, western blot assays showed *MFN1* protein levels to be significantly downregulated in the high glucose and high palmitic acid groups (reduced to 49% and 35%, respectively) (*p* < 0.05) (Figs. 2B, 2C) (raw data is available as Dataset 4). Cells in high glucose and high palmitic acid had significantly higher *DRP1* expression than those in the normal groups (the mRNA of *DRP1* was upregulated to 4.23-fold in the HG group and 5.06-fold in the HF group, Fig. 2A; the protein of *DRP1* was upregulated to 3.18-fold in the HG group and 3.95-fold in the HF group, Figs. 2B, 2F) (Raw data is available as Dataset 3 and Dataset 4) (*p* < 0.01). No significant changes in *MFN2, OPA1* and *FIS1*, whether mRNA levels or protein levels, were seen in cells with high glucose or high palmitic acid treatment (Figs. 2A, 2B, 2D, 2E, 2G) (raw data is available as Dataset 3 and Dataset 4). The results showed that high glucose and high fat inhibited mitochondrial fusion and promoted mitochondrial fission.

**Figure 3:**
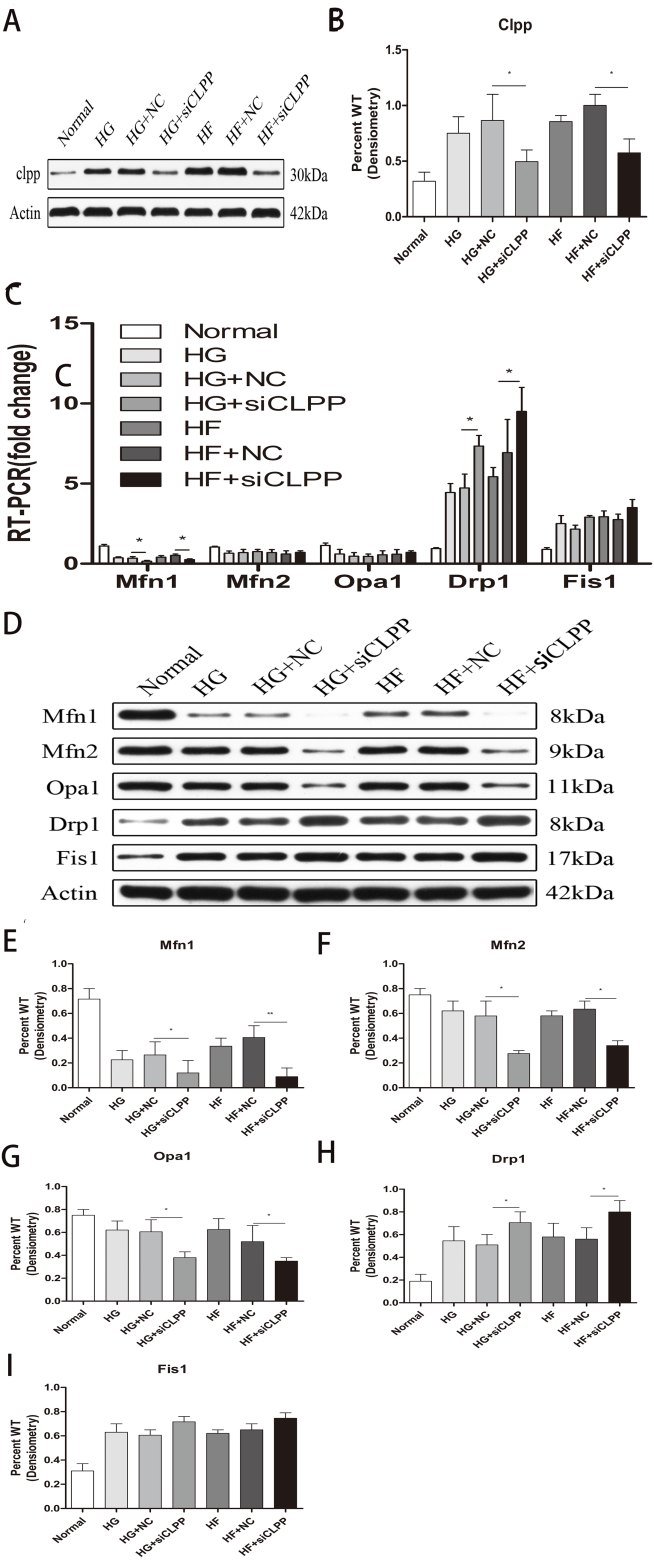
Effect of CLPP siRNA on expression of mitochondrial dynamics-related proteins. (A) Western blot showed CLPP siRNA reduced the expression of CLPP protein. (B) Statistical analysis of the expression of mitochondrial dynamics-related proteins detected by western blot in cells as described in A. RT-qPCR (C) and western blot (D) were used to detect mitochondrial dynamics-related protein expression after CLPP siRNA treatment. (E–I) Statistical analysis of the expression of mitochondrial dynamics-related proteins detected by western blot in cells as described in D. *p < 0.05, **p < 0.01.

### *CLPP* can alleviate the increase of fission and the decrease of fusion induced by high glucose and high fat

*CLPP* siRNA reduced the expression of *CLPP* protein (HG+si*CLPP* and HF+si*CLPP* groups compared with HG+NC and HF+NC groups showed a decrease of 38% and 50%, respectively) (*p* < 0.05) (Figs. 3A,  3B) (raw data is available as Dataset 6). As shown by qRT-PCR analysis, *CLPP* siRNA suppressed expression of *MFN1* (HG+si*CLPP* and HF+si*CLPP* groups compared with HG+NC and HF+NC groups showed a decrease of 52% and 37%, respectively), and enhanced expression of *DRP1* (HG+si*CLPP* and HF+si*CLPP* groups compared with HG+NC and HF+NC groups showed an increase of 68% and 35%, respectively) (*p* < 0.05) (Fig. 3C) (raw data is available as Dataset 5. No significant changes in *MFN2, OPA1* and *FIS1* were seen (Fig. 3C) (raw data is available as Dataset 5. As shown by western blotting analysis, *MFN1* protein levels were decreased by 54% (HG+si*CLPP*) and 77% (HF+si*CLPP*), *MFN2* protein levels were decreased by 53% (HG+si*CLPP*) and 41% (HF+si*CLPP*), *OPA1* protein levels were decreased by 37% (HG+si*CLPP*) and 32% (HF+si*CLPP*), and *DRP1* protein levels were increased by 38% (HG+si*CLPP*) and 43% (HF+si*CLPP*) (*p* < 0.05) (Figs. 3D, 3E, 3F, 3G, 3H) (raw data is available as Dataset 7). No significant change in *FIS1* protein levels was seen (Fig. 3I). As the results showed, *CLPP* siRNA significantly upregulated expression of proteins in mitochondrial fission and downregulated expression of proteins in mitochondrial fusion in the gene-transfected Min6 cells. This implied that the *CLPP* pathway can alleviate the increase of mitochondrial fission and the decrease of mitochondrial fusion induced by high glucose and high fat. The *CLPP* pathway played a role in maintaining the dynamic stability of mitochondrial fusion and fission.

### Expression of *PINK1* and *PARKIN* was highly upregulated in the gene-transfected Min6 cells

**Figure 4:**
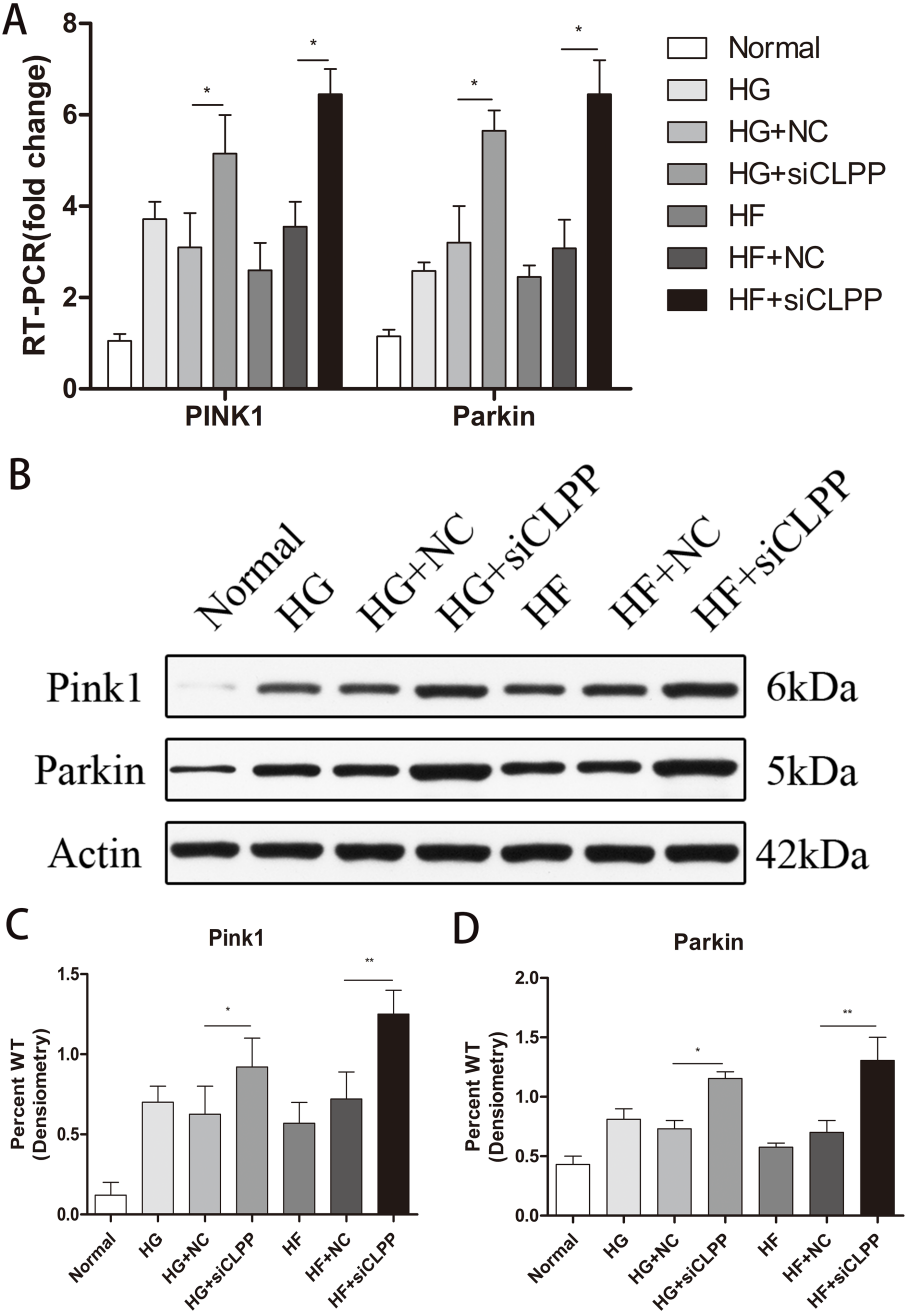
Effect of CLPP siRNA on the expression of mitochondrial autophagy factor PINK1 and PARKIN. (A) Gene expression levels of PINK1 and PARKIN were detected by RT-qPCR. (B) Protein expression of PINK1 and PARKIN was determined by western blot. (C–D) Statistical analysis of the expression of mitochondrial autophagy factor PINK1 and PARKIN detected by western blot in cells as described in B. *p < 0.05, **p < 0.01.

To verify the effects of *CLPP* on mitophagy, we used qRT-PCR and western blot analysis to confirm the alteration in expression of *PINK1* and *PARKIN* in the gene-transfected Min6 cells. In the HG+si*CLPP* and HF+si*CLPP* groups, the mRNA of *PINK1* was upregulated to 1.65-fold and 2.01-fold, respectively, and that of *PARKIN* was upregulated to 1.78-fold and 1.99-fold, respectively (*p* < 0.05) (Fig. 4A) (Raw data is available as Dataset 8). In the HG+si*CLPP* and HF+si*CLPP* groups, *PINK1* protein was upregulated to 1.47-fold and 1.73-fold, respectively, and *PARKIN* protein was upregulated to 1.58-fold and 1.86-fold, respectively (*p* < 0.05) (Figs. 4B, 4C, 4D) (Raw data is available as Dataset 7). Our results suggested that *CLPP* siRNA may significantly increase the expression of *PINK1* and *PARKIN*. The *CLPP* pathway may alleviate the increase of autophagy induced by high glucose and high fat.

### *CLPP* can alleviate the increase of cell apoptosis induced by high glucose and high fat

**Figure 5:**
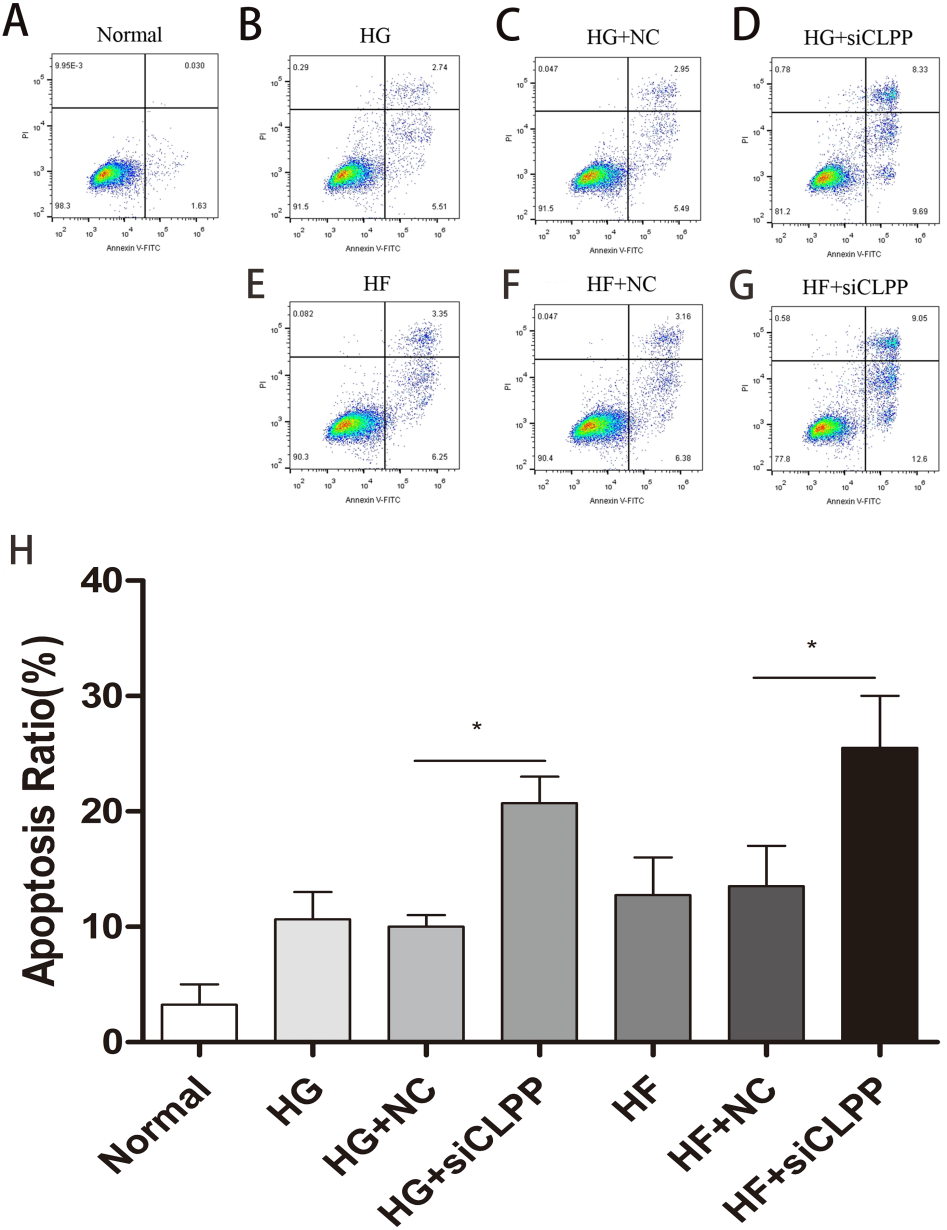
Cell apoptosis was analyzed by Annexin V assays followed by flow cytometry. (A–G) The representative pictures of apoptosis detected by Flow cytometry with FL-1 and FL-2 filters, respectively. Lower left quadrantlive cells (Annexin V/PI), lower right quadrantearly apoptotic cells (Annexin V+/PI), upper right quadrantlate apoptotic cells (Annexin V+/PI+) and upper left quadrantnecrotic cells (Annexin V/PI+). (H) The summary data of apoptosis ratio. *p < 0.05, **p < 0.01.

Annexin-V-FITC/Propidium Iodide flow cytometry was utilized to evaluate the effect of *CLPP* siRNA on Min6 cell apoptosis. The apoptosis proportion (both early and late) rose to 21% from 10% in the HG+si*CLPP* group compared to the HG+NC group. The apoptosis proportion (both early and late) rose to 26% from 14% in the HF+si*CLPP* group compared with just the HF+NC group, when cells were transfected with siRNA-*CLPP* (Figs. 5A–5H) (Raw data is available as Dataset 9. Treatment with *CLPP* siRNA significantly increased apoptosis of Min6 cells. These results showed that *CLPP* could alleviate the increase of cell apoptosis induced by high glucose and high fat.

**Figure 6:**
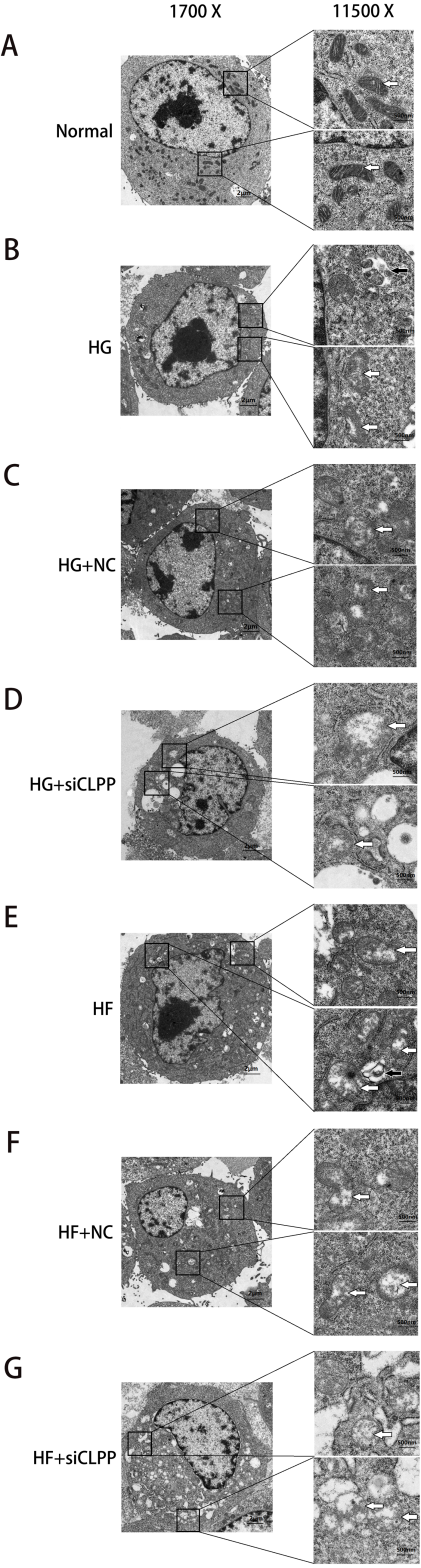
Representative TEM images of mitochondrial ultrastructure at the indicated conditions (A–G). The ultrastructural changes of mitochondria were more obvious in 11,500×images. White arrows indicated mitochondria and black arrows indicated mitophagy. Scale bars of 1,700× indicated 2 µm and scale bars of 11,500× indicated 500 nm.

### *CLPP* may alleviate the structural damage of mitochondria induced by high glucose and high fat

Ultramicrostructure changes of mitochondria were shown in Fig. 6 (Raw data is available as Dataset 10, Dataset 11), as were observed with transmission electron microscopy (TEM). In the Normal group, Min6 cells and mitochondria morphology appeared normal under TEM. The number of mitochondria was more larger (74 mitochondria). The mitochondrion cristae were more and, can be clearly seen along with an intact nuclear membrane. Mitochondria1 matrix was of high density. Swelling mitochondria (the average area of mitochondria was 1.67 ± 0.35 µm^2^ and the area ratio of mitochondria to cell was 1.60) and vacuoles in the cytoplasm were almost absent. In contrast, after high glucose, high glucose+NC, high palmitic acid and high palmitic acid+NC treatment for 48 h, the number of mitochondria was decreased moderately (33, 42, 36 and 32 mitochondria in four groups, respectively). Moreover, the mitochondria were damaged: a small amount of mitochondria swelled and vacuolized. The average area of mitochondria was 3.98 ± 0.77 µm^2^, 4.18 ±1.05 µm^2^, 4.30 ± 0.80 µm^2^, and 4.73 ± 1.36 µm^2^, respectively (compared with the Normal group, *P* < 0.05). The area ratio of mitochondria to cell was3.12, 3.49, 3.66, and 3.97, respectively. The mitochondrial cristae were also broken and less and difficult to identify. Mitochondria1 matrix was of medium density. Fortunately, mitophagy images were captured in HG group and HF group. There were some vacuoles in the cytoplasm. Particularly, in the HG+si*CLPP* and HF+si*CLPP* groups, mitochondria were rare (17 and 16 mitochondria in two groups, respectively), and the mitochondria and nuclei were damaged, including missing cristae and a damaged matrix. Swelling mitochondria were obvious. The average area of mitochondria was 9.23 ± 1.58 µm^2^ and 9.10 ± 1.71 µm^2^, respectively (compared with the HG+NC and HF+NC groups, *P* < 0.05). The area ratio of mitochondria to cell was 8.15 and 7.98, respectively. Mitochondria were more pronounced vesicular in cross section. Mitochondria1 matrix was of low density. There were many and obvious vacuoles in the cytoplasm. Fewer mitochondria were observed, suggesting that more mitochondria may be cleared by autophagy after fission. *CLPP* siRNA aggravated mitochondrial damage induced by high glucose and high fat. These results suggested that the *CLPP* pathway may play a protective role in mitochondrial damage induced by stress (e.g., high glucose and high fat).

**Figure 7:**
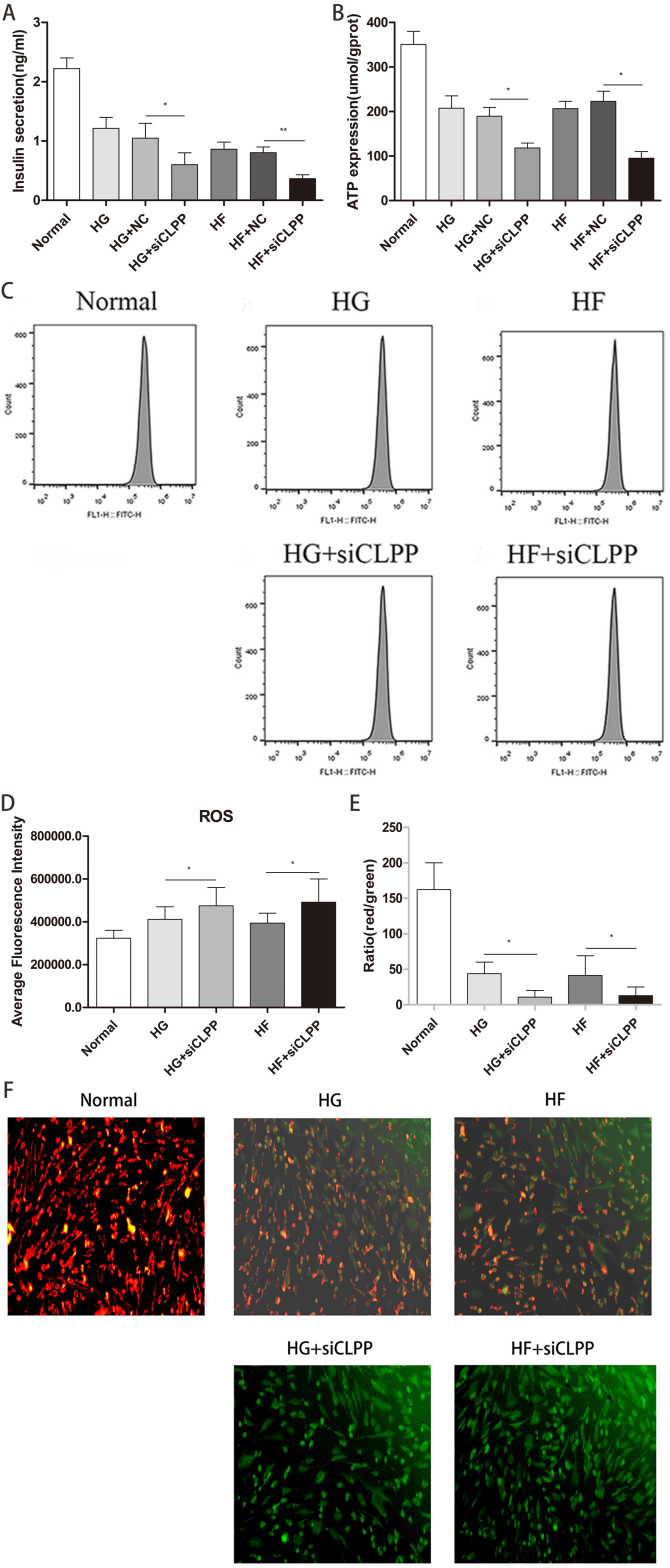
Evaluation of mitochondrial function (Part 1). (A) Effect of CLPP siRNA on mitochondrial secretion of insulin. (B) Effect of CLPP siRNA on mitochondrial ATP production. (C) The representative pictures of ROS detected by Flow cytometry. (D) Comparison of fluorescence intensity of ROS in Flow cytometry. (E) The ratio of red/green fluorescent densities was calculated to evaluate the change of relative mitochondrial membrane potential. (F) Mitochondrial membrane potential fluorescence images visualized by a fluorescence microscope (200×). In cultured MIN6 cells after different treatments, representative images of JC-1 staining were shown CLPP siRNA reduced mitochondrial membrane potential. The green color indicated decreased membrane potential and the red color indicated normal membrane potential.

### Insulin secretion was decreased by knocking down *CLPP*

To study a potential function of *CLPP* in regulating insulin secretion, we assessed the effects of *CLPP* siRNA on insulin secretion in Min6 cells. Our analysis revealed that insulin secretion levels were lower in the HG+si*CLPP* and HF+si*CLPP* groups compared to those of the HG+NC and HF+NC groups (decreased by 43% and 49%, respectively) (*p* < 0.05) (Fig. 7A) (Raw data is available as Dataset 12). The *CLPP* pathway was involved in the regulation of insulin secretion.

### Knocking down *CLPP* reduced ATP secretion

The results showed that *CLPP* siRNA can cause less ATP to be released by Min6 cells in the HG+si*CLPP* and HF+si*CLPP* groups compared with the HG+NC and HF+NC groups (decreased by 38% and 57%, respectively) (*p* < 0.05) (Fig. 7B) (Raw data is available as Dataset 13). This indicated that *CLPP* affected mitochondrial function and further interfered with the ATP pathway in mitochondria.

### ROS generation was increased by knocking down *CLPP*

We then tested the effect of si*CLPP* on high glucose and high fat-induced ROS generation. As shown in Figs. 7C, 7D (Raw data is available as Dataset 14), si*CLPP* increased the high glucose and high fat-induced ROS generation in Min6 cells by 15% and 25%, respectively. These findings suggested that *CLPP* may be a regulator of mitochondrial ROS generation.

### *CLPP* was beneficial to maintaining mitochondrial membrane potential

We examined the effect of *CLPP* siRNA on mitochondrial membrane potential in Min6 cells using JC-1 loading. Figure 7E showed that the density of red color in the Normal group was more higher, indicating that mitochondrial membrane potential was more larger. The density of red color was decreased and the density of green color was increased in the HG and HF groups, which meant that mitochondrial membrane potential was decreased. In the HG+si*CLPP* and HF+si*CLPP* groups, green fluorescence became markedly stronger and red fluorescence was obviously reduced, which meant that mitochondrial membrane potential was more smaller. Similarly, in Fig. 7F the results showed the red/green ratio was more higher in the Normal group, indicating that the mitochondrial membrane potential was more larger, and the middle in HG, HF groups, which meant that the mitochondrial membrane potential was decreased, and more lower in HG+si *CLPP*, HF+si *CLPP* groups compared with HG, HF groups, which meant that the mitochondrial membrane potential was more smaller (red/green ratio was decreased by 75% and 69%, respectively) (*p* < 0.05) (Fig. 7F) (raw data is available as Dataset 15).

### Mito-Tracker Red CMXRos Staining

**Figure 8:**
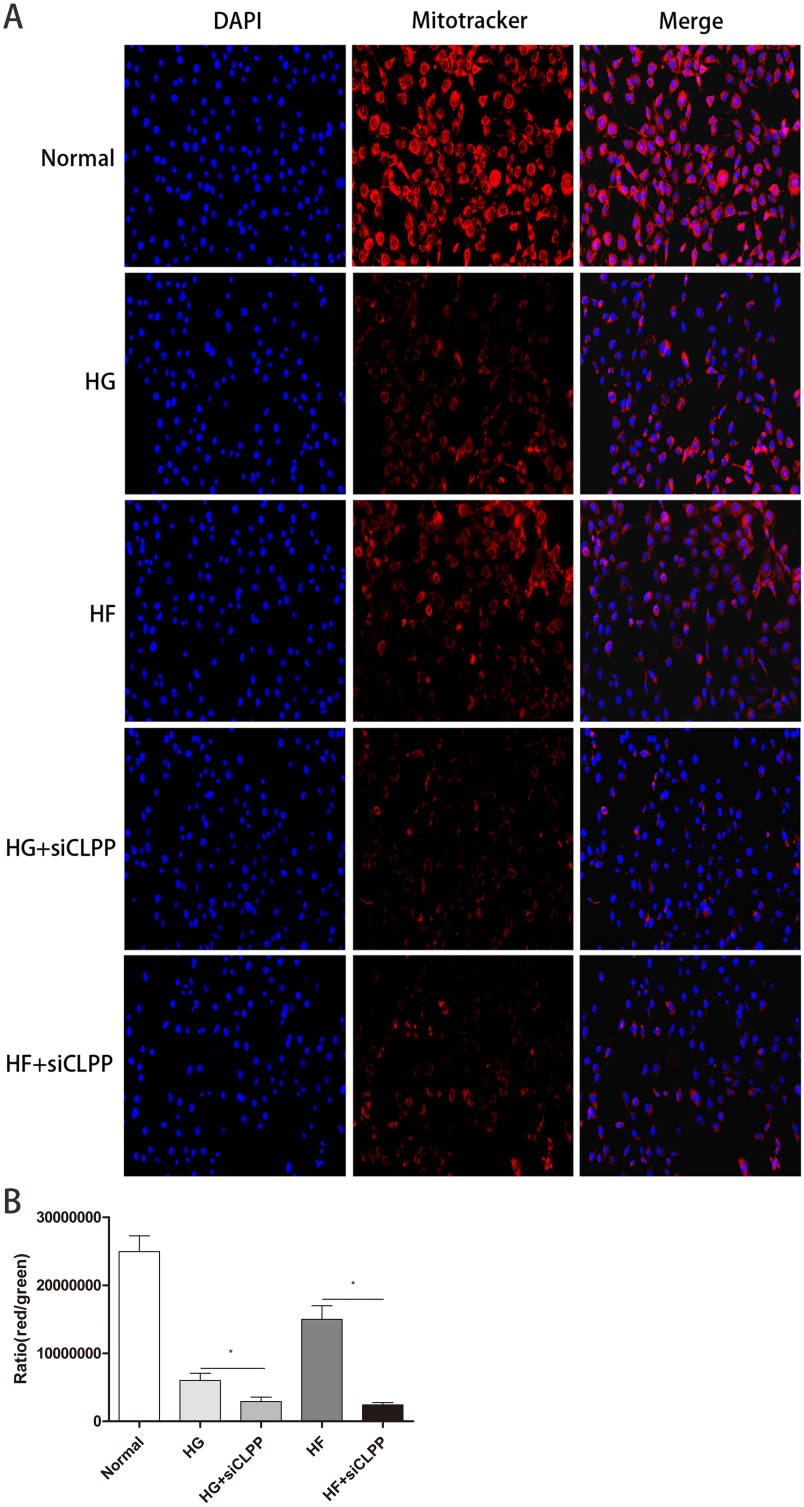
Evaluation of mitochondrial function (Part 2). (A) The fluorescence intensity of bioactive mitochondria was observed by fluorescence microscopy (200×) after Mito-Tracker Red CMXRos staining. Blue color: nuclus stained with DAPI, red color: mitochondria stained with Mito-Tracker Red CMXRos. (B) IOD value of red light intensity was calculated after Mito-Tracker Red CMXRos staining. *p < 0.05, **p < 0.01.

The number of mitochondria with more detailed statistics for the various treatment groups was determined using Mito-Tracker Red CMXRos staining. Intracellular mitochondria were stained red and nucleus were blue (Fig. 8A) (raw data is available as Dataset 16). The intensity of red light correlated with the number of bioactive mitochondria. After Mito-Tracker Red CMXRos staining, the stronger the intensity of red light, the more the number of bioactive mitochondria. Results showed that the number of bioactive mitochondria were fewer in the HG+si*CLPP* and HF+si*CLPP* groups compared to those in the HG and HF groups. IOD values reflecting red light intensity were lower in the HG+si*CLPP* and HF+si*CLPP* groups than in the HG and HF groups (IOD values were decreased by 53% and 86%, respectively) (*p* < 0.05) (Fig. 8B).

## Discussion

Our data demonstrated that treating cells with high glucose and high fat for 48 h caused upregulation of the mitochondrial protease, *CLPP*. Furthermore, the association between high glucose or high fat and upregulation of *CLPP* was found to be time and dose dependent. However, the expression of *CLPP* was insignificant between 1 mmol/l palmitic acid group and 0.5 mmol/l palmitic acid group at all-time points. This may be associated with a significant increase in Min6 cell death after 1 mmol/l palmitic acid treatment as compared to 0.5 mmol/l palmitic acid treatment, which results in a decrease of *CLPP* production. Time and dose dependence indicated that the activation of the mtUPR requires relatively intense environmental stress. In worms, for example, the activation of *CLPP* genes require sustained perturbation of mitochondrial protein-folding homeostasis over 3 days (Haynes et al., 2007). Upon environmental insults (for example, nutrient excess), mitochondria harbor a large set of proteases involved in quality control, including *CLPP* (Becker et al., 2018). High glucose and high fat may cause the increase and accumulation of unfolded and misfolded proteins in mitochondria, which further can result in a mito-nuclear protein imbalance, activating the mtUPR.

*CLPP* plays a critical role in high-fat stress conditions (Becker et al., 2018). Mammalian *CLPP* substrates include several metabolic enzymes involved in fatty acid β-oxidation (FAO) (Szczepanowska et al., 2016). It can be speculated that the expression of *CLPP* in the mitochondrial *CLPP*-FAO pathway increases as a result of higher concentrations and longer periods of high fat treatment given, so that *CLPP* can participate more effectively in fatty acid metabolism. It should be noted that different types of fatty acids have different effects on *CLPP*. Palmitic acid, as a saturated fatty acid, increase the expression of *CLPP* in our experiments, whereas in white adipose tissue, expression of *CLPP* is induced by unsaturated fatty acids, with saturated fatty acids reducing the expression of *CLPP* (Bhaskaran et al., 2017). The effects of fatty acids on *CLPP* may be tissue specific.

*CLPP* is involved in the process of glucose metabolism. Knockdown of *CLPP* can lead to the improvement of glucose metabolism, which could be attributed to the increased expression of *GLUT4* in different tissues of *CLPP*-deficient mice. In skeletal muscle, the increase of *GLUT4* level likely relates to the activation of *AMPK* (Kurth-Kraczek et al., 1999). High glucose concentration in Min6 cells mimicks hyperglycemia in vivo for inducing UPR signaling (Wang et al., 2015). In our research, high glucose can induce an increase of *CLPP* expression. However, to address whether this is achieved through the participation of *CLPP* in the process of glucose metabolism requires further research and experimentation.

In our experiments, after cells were treated with high glucose and high fat, mitochondrial fusion-related protein *MFN1* was decreased and mitochondrial fission-related protein *DRP1* was increased, indicating that mitochondrial fusion was decreased, and mitochondrial fission was increased under stress. However, this phenomenon was not obvious after 24 h of high glucose and high fat treatment. Our findings generally agreed with previous studies on the strong relationship between energy demand and supply balance and mitochondrial morphology (Gomes, Di Benedetto & Scorrano, 2011; Molina et al., 2009). However, there are inconsistent conclusions about the specific changes of mitochondrial dynamics-related protein expression after high glucose and high fat treatment. It seems likely that different cell types harness these processes in distinct ways which are best suited to their cellular stress. A high glucose culture is induced to undergo mitochondrial fission in cultured primary neonatal rat cardiomyocytes by decreasing the expression of *MFN1* and *OPA1* and increasing the expression of *MFN2, FIS1*, and *DRP1* (Yu et al., 2017). In INS1 cells incubated with 20 mmol/l glucose, expression of proteins (*MFN1, MFN2, OPA1, FIS1*, and *DRP1*) is significantly reduced, with the greatest reduction for *FIS1* (Schultz et al., 2016). High glucose induces an increase in *DRP1* in podocytes (Ma et al., 2016). High glucose and high palmitate increase expression of *FIS1* and p-*DRP1/DRP1* and decrease expression of *MFN2* (Liu et al., 2017). In animal experiments, *MFN2, OPA1*, and *FIS1* demonstrate significant differences between a high-fat diet group and a normal diet group (Chen et al., 2018). The mechanisms underlying high glucose and high fat-induced alterations in mitochondrial fusion and fission related proteins are still not fully understood.

The duration of mitochondrial fusion and fission is very short. Fusion takes between 15.0 and 17.5 s, while mitochondrial fission takes between 35.0 and 67.5 s (Bereiter-Hahn & Voth, 1994; Haynes et al., 2007). Therefore, it is difficult to capture instantaneous images of mitochondrial fusion and fission through electron microscopy. The overall trend of mitochondrial fusion and fission can be reflected by the difference in the number of mitochondria and microstructure of mitochondria between different groups. Transmission electron microscopy analysis revealed that the morphological changes of mitochondria fit with the trend of the expression of mitochondrial dynamics-related proteins in the case of environmental stress. After high glucose and high fat treatment, the number of mitochondria and cristae was decreased, suggesting that the balance between fusion and fission was broken (i.e., fission was increased, and fusion was decreased), and indicating that mitochondria showed a distinct pattern of damage under cellular stress.

The role of *CLPP* in mitochondrial dynamics changes mediated by high glucose and high fat has not yet been reported. Wang et al. examined whether an environment (H2O2 treatment) with impaired energy metabolism affects mitochondrial dynamics in *CLPP*^−/−^ mice oocytes. Findings revealed that fusion related proteins (MFN1, MFN2, OPA1) were decreased, while the expression of DRP1, which mediates fission, was unchanged. Mitochondrial dynamics seem to be severely affected in *CLPP*^−/−^ oocytes. Morphometric analysis using electron microscopy found that *CLPP*^−/−^ oocytes had shorter and smaller mitochondria, suggesting decreased fusion (Wang et al., 2018; Westermann, 2010). In our study, we sought to understand the role of *CLPP* in the effects of high glucose and high fat on mitochondrial dynamics. To achieve this, we knocked down the expression of CLPP. It is likely that the consequent reduced expression of *CLPP* contributed to the downregulation of transcripts coding for fusion proteins and the increase of fission-related proteins. These findings highlighted the importance of CLPPs proteolytic activity in mitochondrial dynamics. A possible mechanism is that knockdown of *CLPP* interferes with the mtUPR. Under high glucose and high fat stress, unfolded and misfolded proteins cannot be cleared by the mtUPR and accumulate in the mitochondria, resulting in mitochondrial fission increasing and fusion decreasing. This effect is manifested not only in the expression of mitochondrial dynamics-related proteins but also in the number and ultrastructure of mitochondria analyzed by electron microscopy. Due to increased fission and reduced fusion, the number of mitochondria is decreased. In addition, with the decrease in the number of mitochondria, mitochondrial damage in *CLPP* knock-down cells is more evident, including mitochondrial edema and cristae disappearance. The trend in the changes of mitochondrial dynamics-related proteins was the same as that of mitochondrial morphology by electron microscopy analysis. It may be considered that CLPP affects mitochondrial dynamics by affecting the mtUPR. CLPP-1 (RNAi) can perturb mitochondrial morphology, a finding consistent with higher level of the mtUPR in the mitochondria of animals with CLPP deficiency (Haynes et al., 2007). An important question posed by the research is, regarding the impact of CLPP on mitochondrial dynamics, which is the main influencer? Fusion or fission? At present, fission is considered more important. The mtUPR can induce mitochondrial fission (Aldridge, Horibe & Hoogenraad, 2007). In C2C12 cells lacking CLPP, the increase in the presence of DRP1 was related to reduction in mitochondrial size (Deepa et al., 2016). However, it is not clear whether CLPP directly controls mitochondrial dynamics. What are the specific intermediate links between CLPP and mitochondrial dynamics-related proteins? Future studies in this area will, again, help clarify any potential impact.

The mtUPR affects multiple mitochondrial functions including mitophagy. In the case of an accumulation of damaged polypeptides, if the damage is too severe and dysfunctional mitochondria accumulate, the cell will specifically remove these organelles by mitophagy. The pathway regulating mitophagy mediated by *PINK1* and *PARKIN* is present in many metazoans (Eiyama & Okamoto, 2015). Our experiment showed that the expression of *PINK* and *PARKIN* was increased after knockdown of *CLPP* in the conditions of high glucose and high fat. This means that the mtUPR acts as a protective factor, and if this process is impaired, such as by knocking down *CLPP*, damage to the mitochondria increases and eventually mitophagy increases.

*CLPP* also affects mitochondrial function. Knocking down *CLPP* leads to a further decrease in mitochondrial synthesis of ATP, a further reduction of insulin secretion in cells and a further increase in ROS synthesis. These results also show that mtUPR is closely related to mitochondrial function. Several previous studies supported a role for *CLPP* in the maintenance of mitochondrial function. For example, shRNA-mediated *CLPP* silencing in OCIAML2 cell lines showed increased generation of ROS (Bhaskaran et al., 2018). *CLPP*-deficient cells had increased mitochondrial ROS levels (Deepa et al., 2016). Decline in *CLPP* in C2C12 cells resulted in elevated production of ROS (Deepa et al., 2016). The ROS levels were increased in gWAT of *CLPP*^−∕−^ mice (Fisler & Warden, 2006).

The most outstanding feature of min6 cell (mice pancreatic β-cell line) is that they produce a large amount of insulin. ATP is an important signalling molecule, which regulates a production of insulin in min6 cell (Kowal, Yegutkin & Novak, 2015). When the lack of protease *CLPP* in mitochondria leads to the increase of unfolded and misfolded proteins, ATP generation is interfered and then reduced, and the production of insulin in min6 cells will be reduced accordingly. Whether CLPP affects insulin production through ATP pathway and whether CLPP affects ATP production by affecting the respiratory chain in mitochondria need further study to confirm. Studies by Bhaskaran et al. found circulating levels of insulin were reduced by 68% in *CLPP*^−∕−^ mice compared to WT mice (Bhaskaran et al., 2018). However, some experiments showed conflicting results. *CLPP*^−∕−^ mice had elevated insulin sensitivity and did not exhibit lower circulating insulin levels (Becker et al., 2018). The effect of CLPP on insulin needs further experimental confirmation.

## Conclusions

As an important protease in the mtUPR, *CLPP* plays an important role in the morphology and function of mitochondria under cellular stress (such as high glucose and high fat conditions). There is a very close relationship among the mtUPR, mitochondrial dynamics, mitochondrial morphology, and mitochondrial function.

##  Supplemental Information

10.7717/peerj.7209/supp-1 Dataset 1 RT-PCR detection of *CLPP* expression at 24 h, 36 h, 48 h after different concentrations of glucose and palmitic acid

10.7717/peerj.7209/supp-2 Dataset 2 RT-PCR results of high glucose (33.3 mmol/l) and high fat (0.5 mmol/l) treatment for 48h

10.7717/peerj.7209/supp-3 Dataset 3 RT-qPCR showed the expression levels of mitochondrial dynamics-related proteins

10.7717/peerj.7209/supp-4 Dataset 4 Effect of CLPP siRNA on the expression of mitochondrial autophagy factor PINK1 and PARKIN

10.7717/peerj.7209/supp-5 Dataset 5 RT-qPCR was used to detect mitochondrial dynamics-related protein expression after *CLPP* siRNA treatment

10.7717/peerj.7209/supp-6 Dataset 6 RT-qPCR was used to detect mitochondrial dynamics-related protein expression after *CLPP* siRNA treatment

10.7717/peerj.7209/supp-7 Dataset 7 Western blot was used to detect mitochondrial dynamics-related protein expression after *CLPP* siRNA treatment

10.7717/peerj.7209/supp-8 Dataset 8 Gene expression levels of *PINK1* and *PARKIN* were detected by RT-qPCR

10.7717/peerj.7209/supp-9 Dataset 9 Representative pictures of apoptosis detected by flow cytometry

10.7717/peerj.7209/supp-10 Dataset 10 Representative TEM images of mitochondrial ultrastructure at the indicated conditions

10.7717/peerj.7209/supp-11 Dataset 11 The area of mitochondria and the total cell were circled and calculated by AOI tool of Image Pro Plus 6.0 software

10.7717/peerj.7209/supp-12 Dataset 12 Effect of *CLPP* siRNA on mitochondrial secretion of insulin

10.7717/peerj.7209/supp-13 Dataset 13 Effect of *CLPP* siRNA on mitochondrial ATP production

10.7717/peerj.7209/supp-14 Dataset 14 Comparison of fluorescence intensity of ROS in flow cytometry

10.7717/peerj.7209/supp-15 Dataset 15 The ratio of red/green fluorescent densities was calculated to evaluate the change of relative mitochondrial membrane potential

10.7717/peerj.7209/supp-16 Dataset 16 The fluorescence intensity of bioactive mitochondria was observed by fluorescence microscopy (200 ×) after Mito-Tracker Red CMXRos staining

## Additional Information and Declarations

### Data Availability

The following information was supplied regarding data availability: Raw data is available in the Supplemental Files.
