# Supplementary material for: Mitochondrial unfolded protein response gene CLPP changes mitochondrial dynamics and affects mitochondrial function
Source: PeerJ. 2019 Jul 2;7:e7209. doi: 10.7717/peerj.7209 (PMC6611452; doi:10.7717/peerj.7209)
Supplement: Dataset 9 [file peerj-07-7209-s009.tif › 20181009min6╡≥═÷╟≈╩╞.pdf]

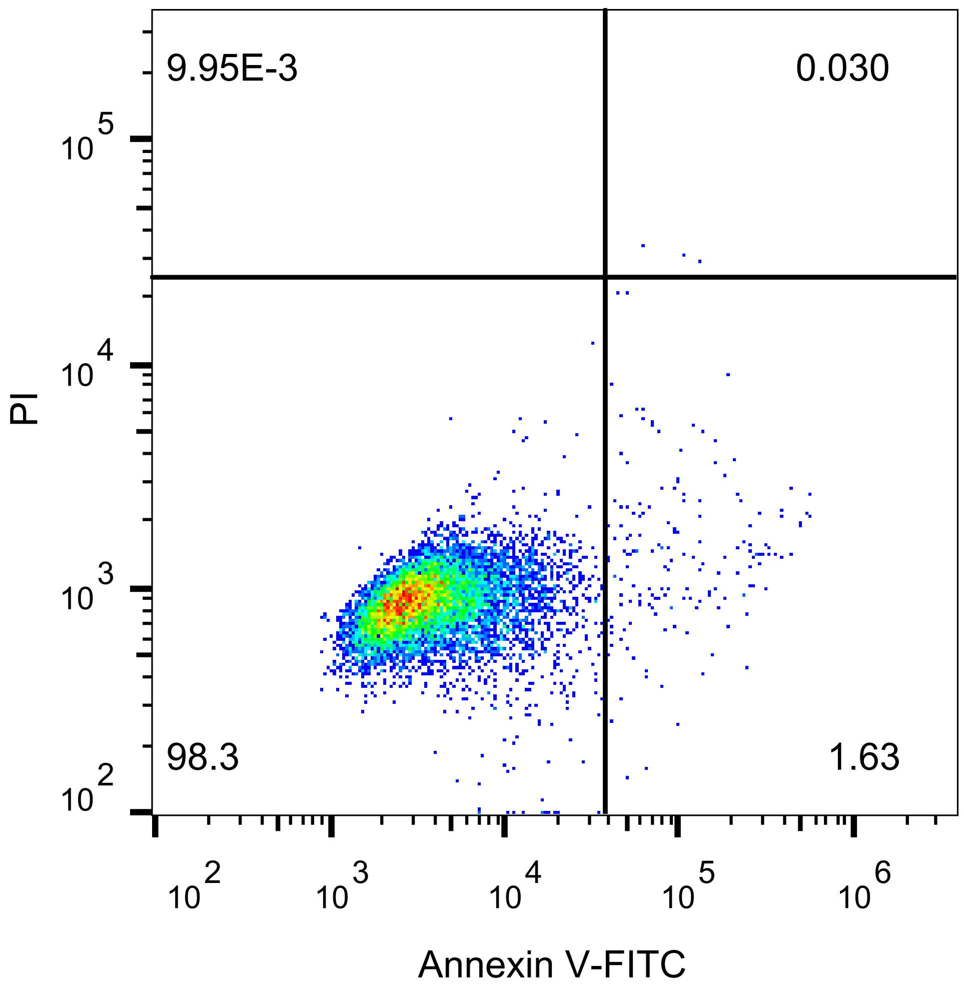

LD-1.fcs

FSC-A, FSC-Width subset

10047

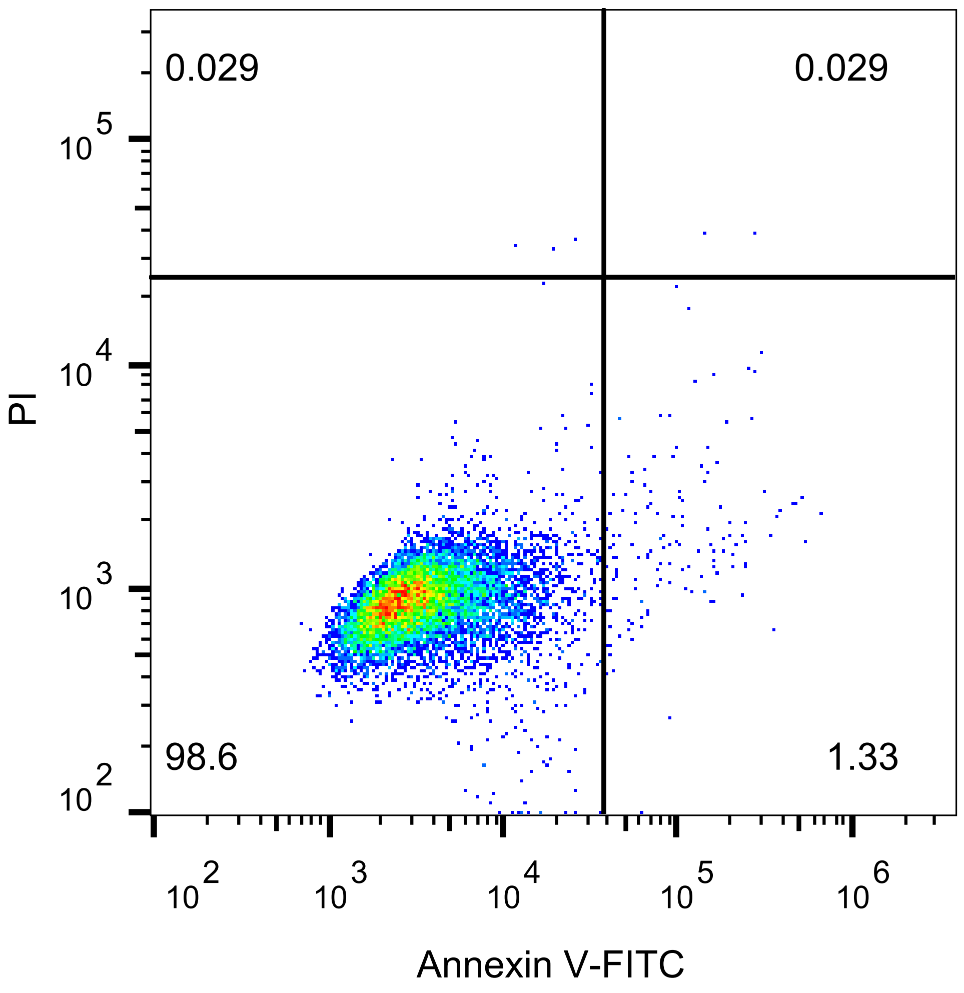

LD-2.fcs

FSC-A, FSC-Width subset

10455

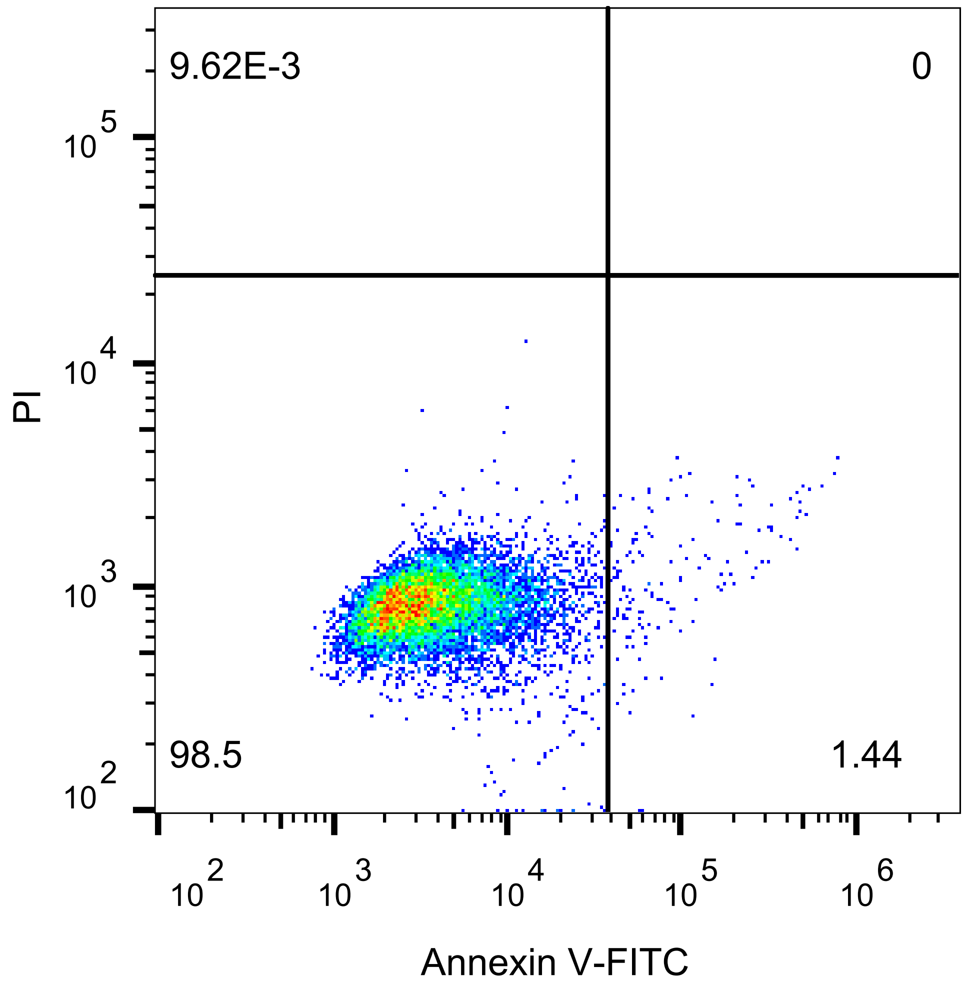

LD-3.fcs
FSC-A, FSC-Width subset
10400

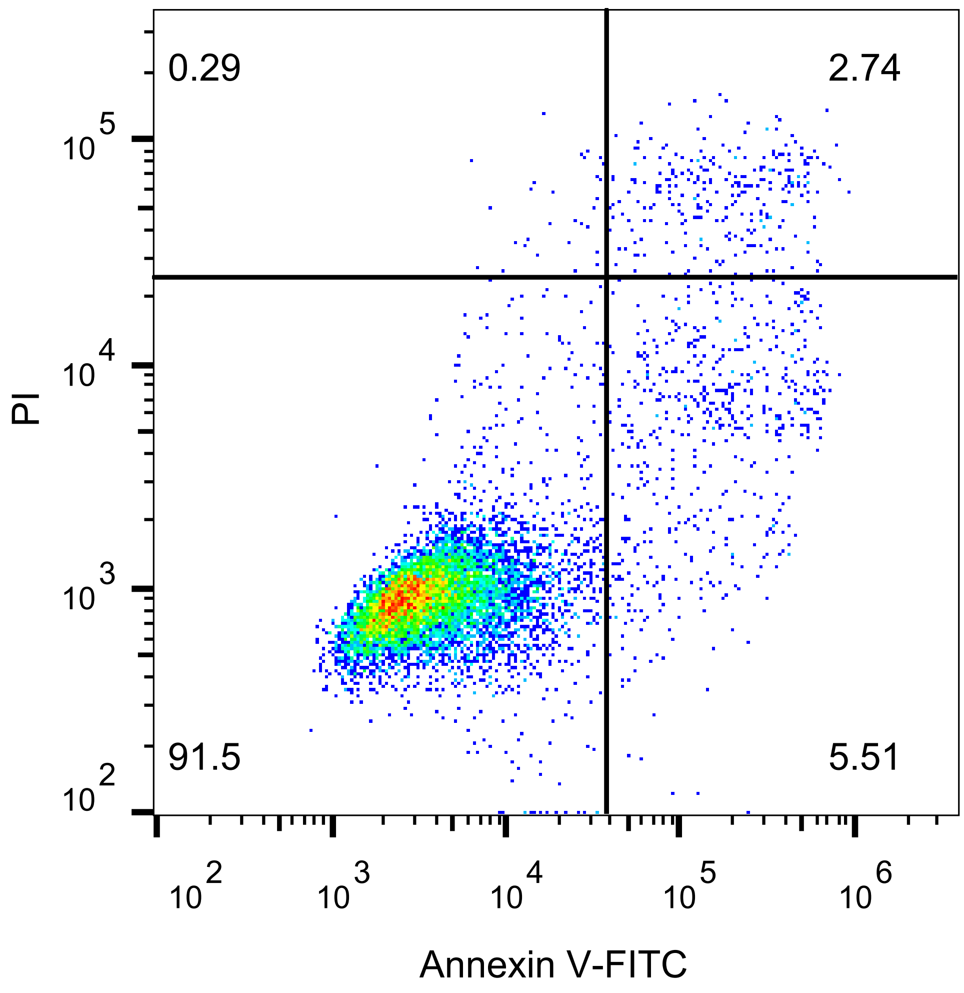

HD-1.fcs

FSC-A, FSC-Width subset

10265

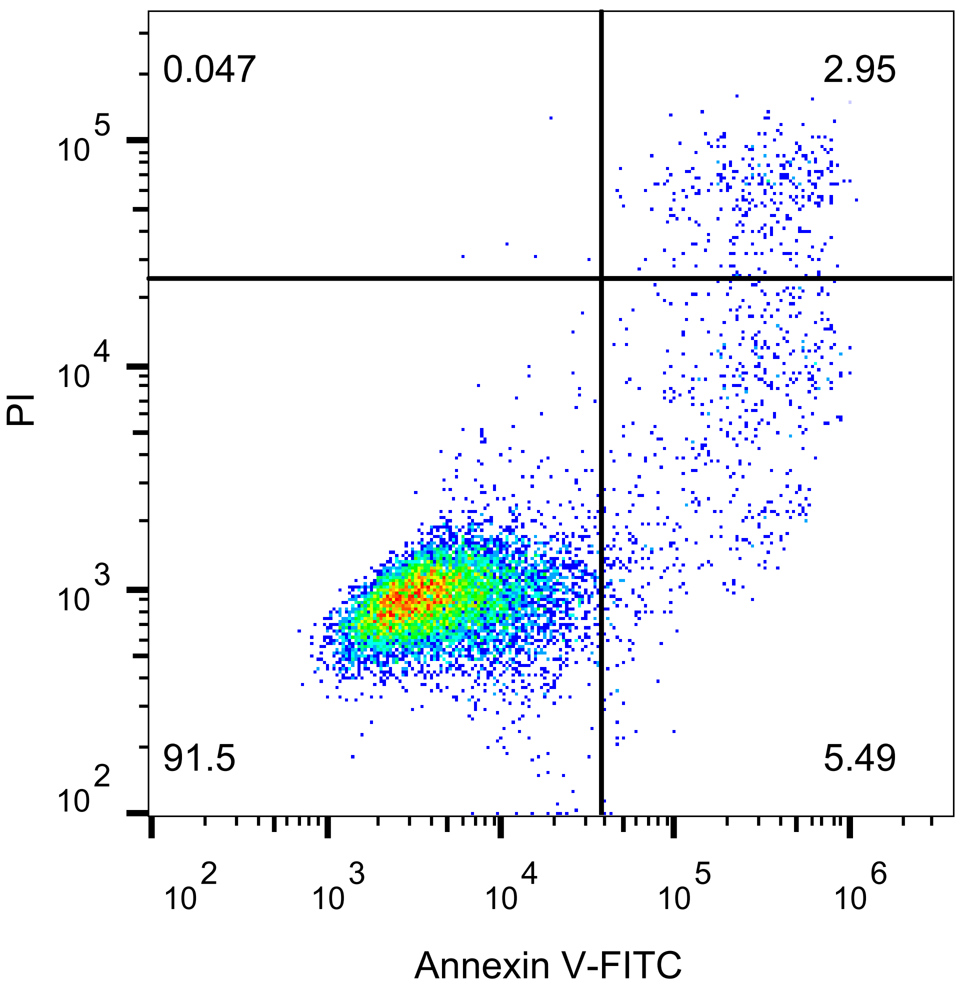

HD-2.fcs

FSC-A, FSC-Width subset

10742

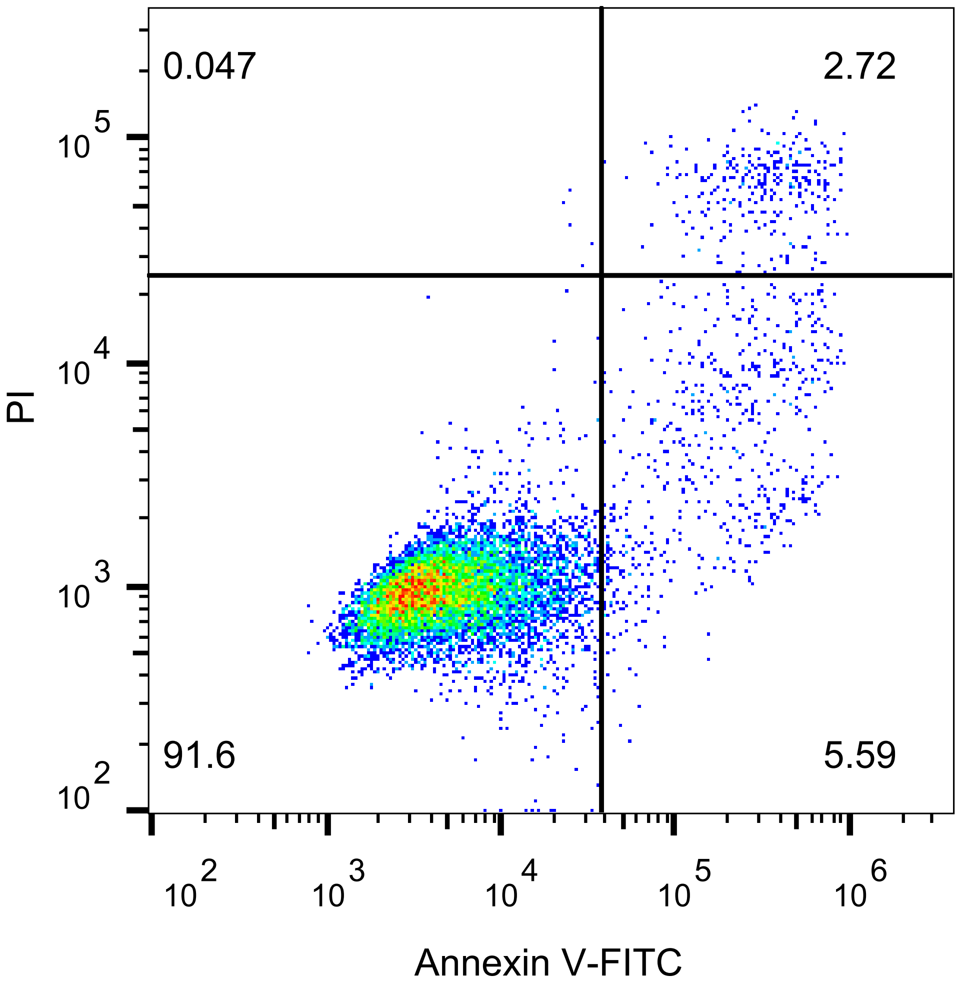

HD-3.fcs

FSC-A, FSC-Width subset

10547

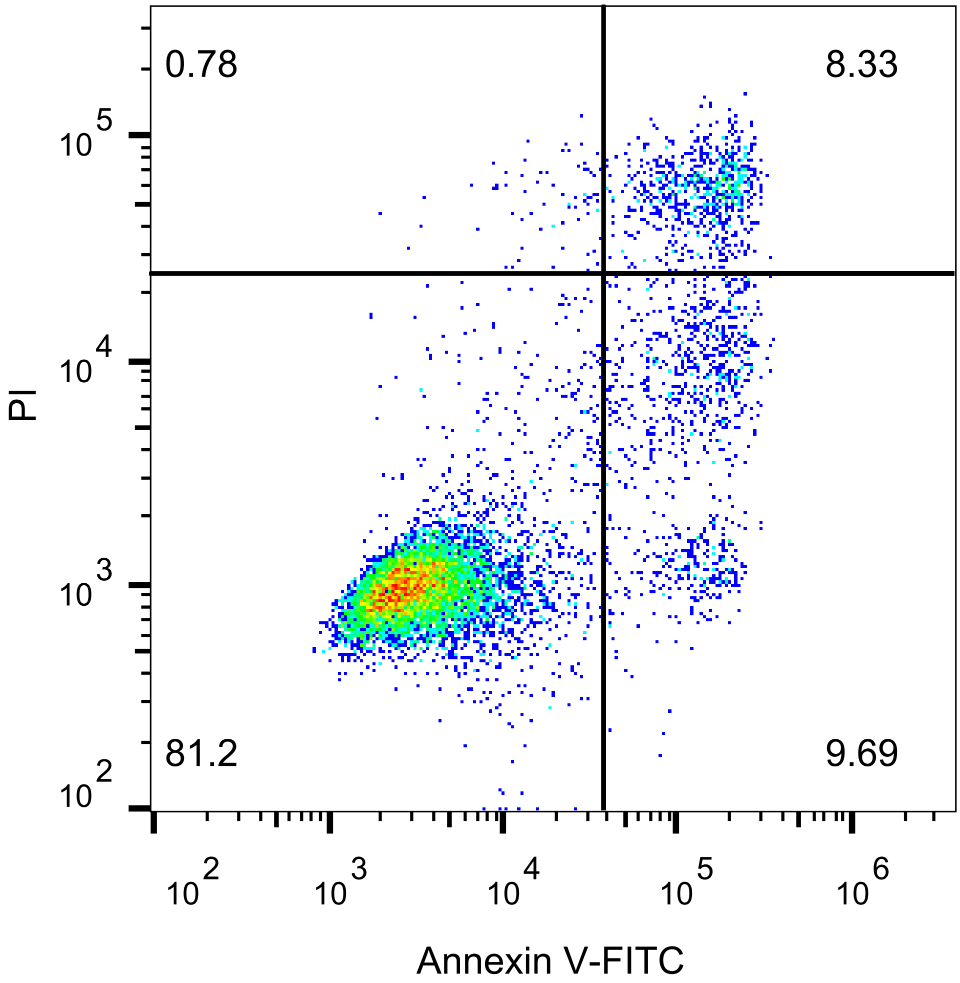

H-siclpp-1.fcs
FSC-A, FSC-Width subset
9475

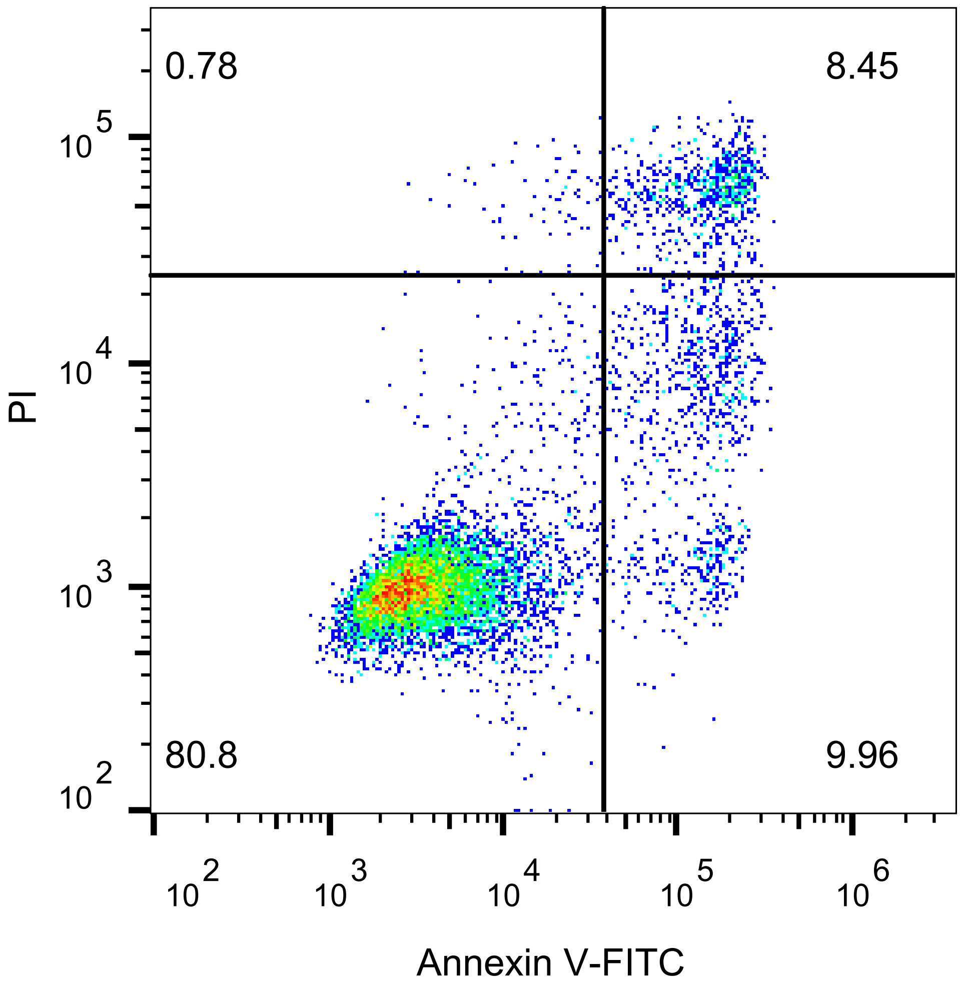

H-siclpp-2.fcs
FSC-A, FSC-Width subset
9247

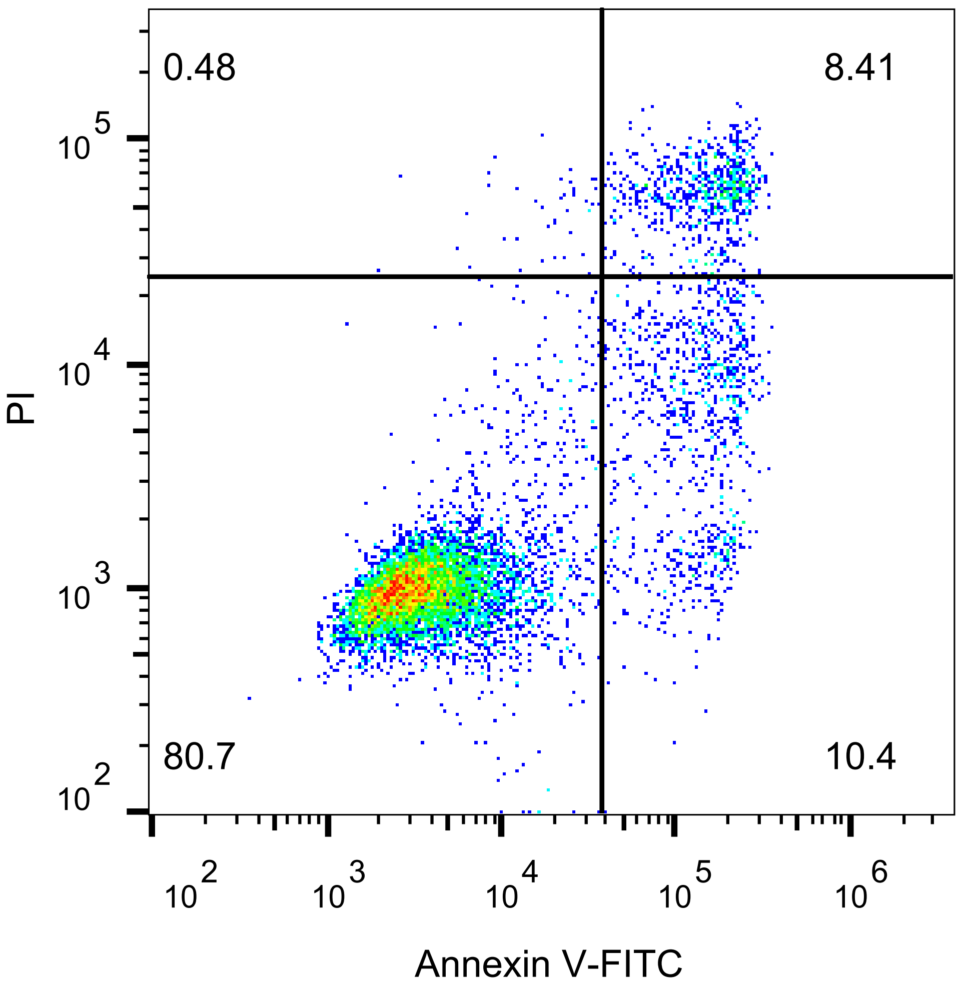

H-siclpp-3.fcs
FSC-A, FSC-Width subset
9151

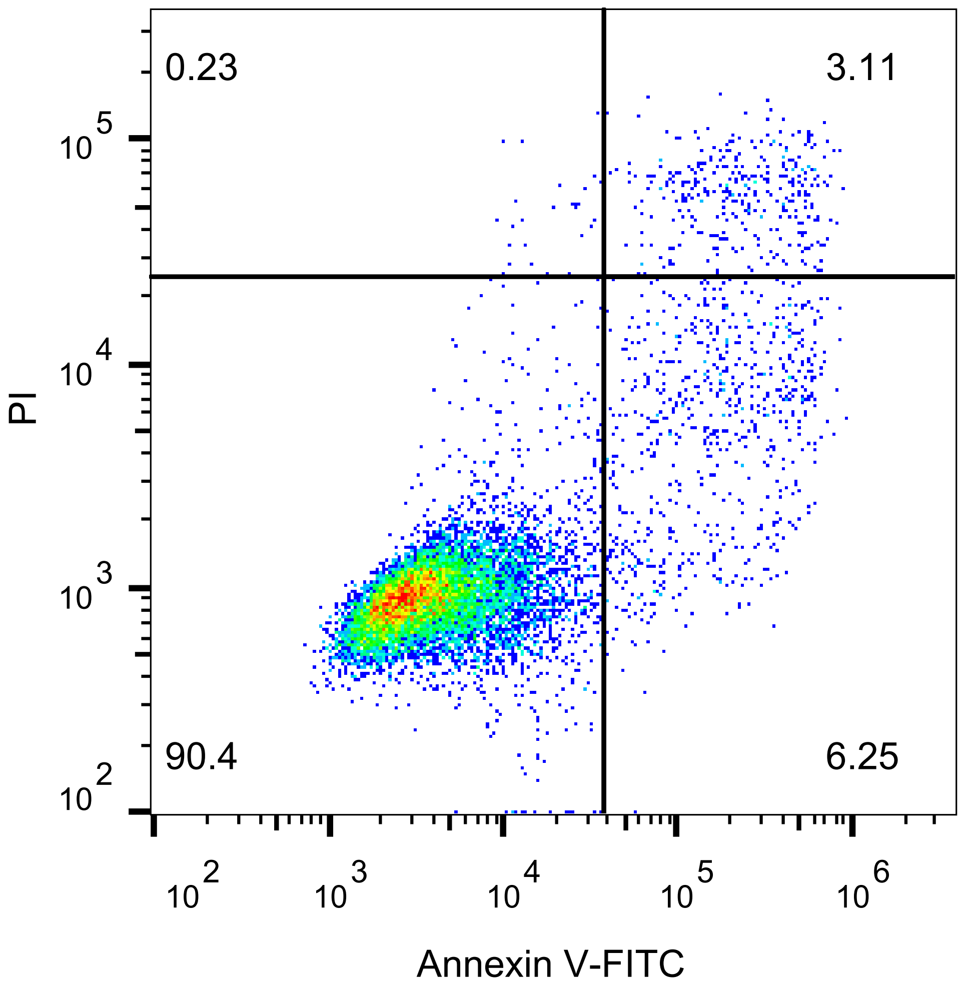

HF-1.fcs

FSC-A, FSC-Width subset

11127

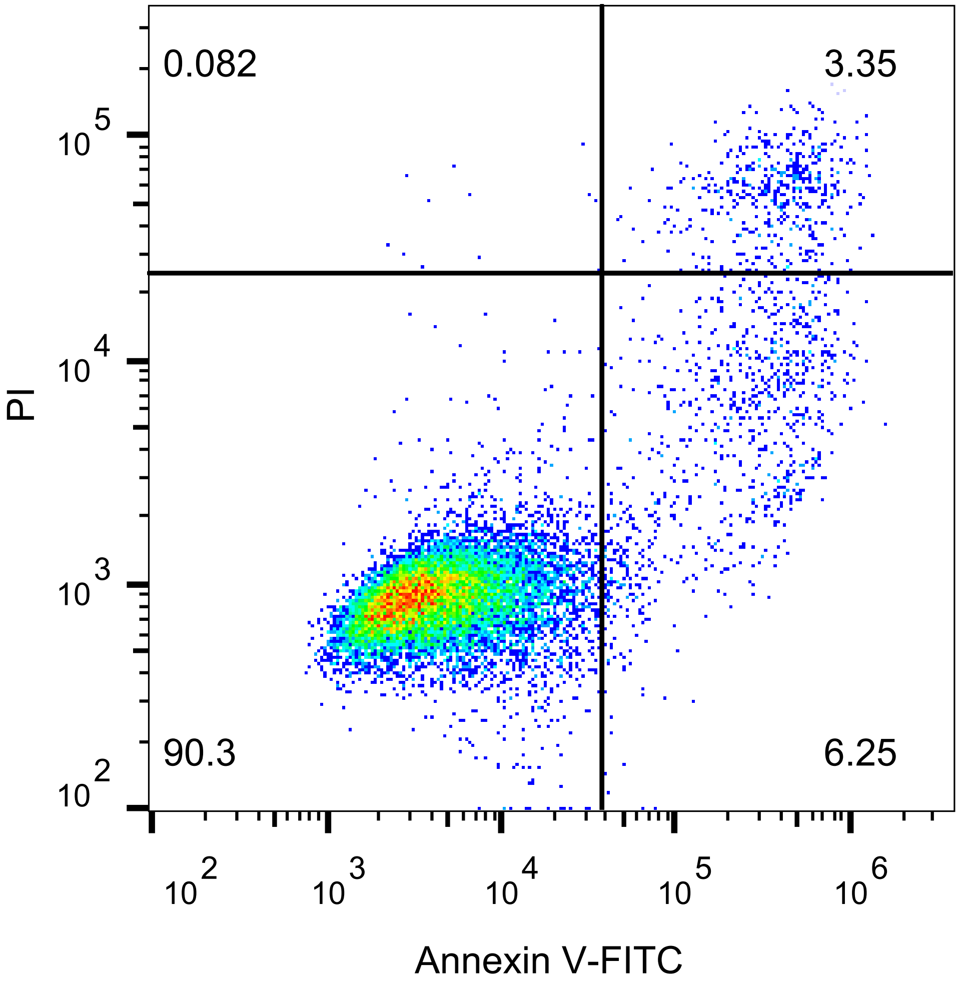

HF-2.fcs
FSC-A, FSC-Width subset
14648

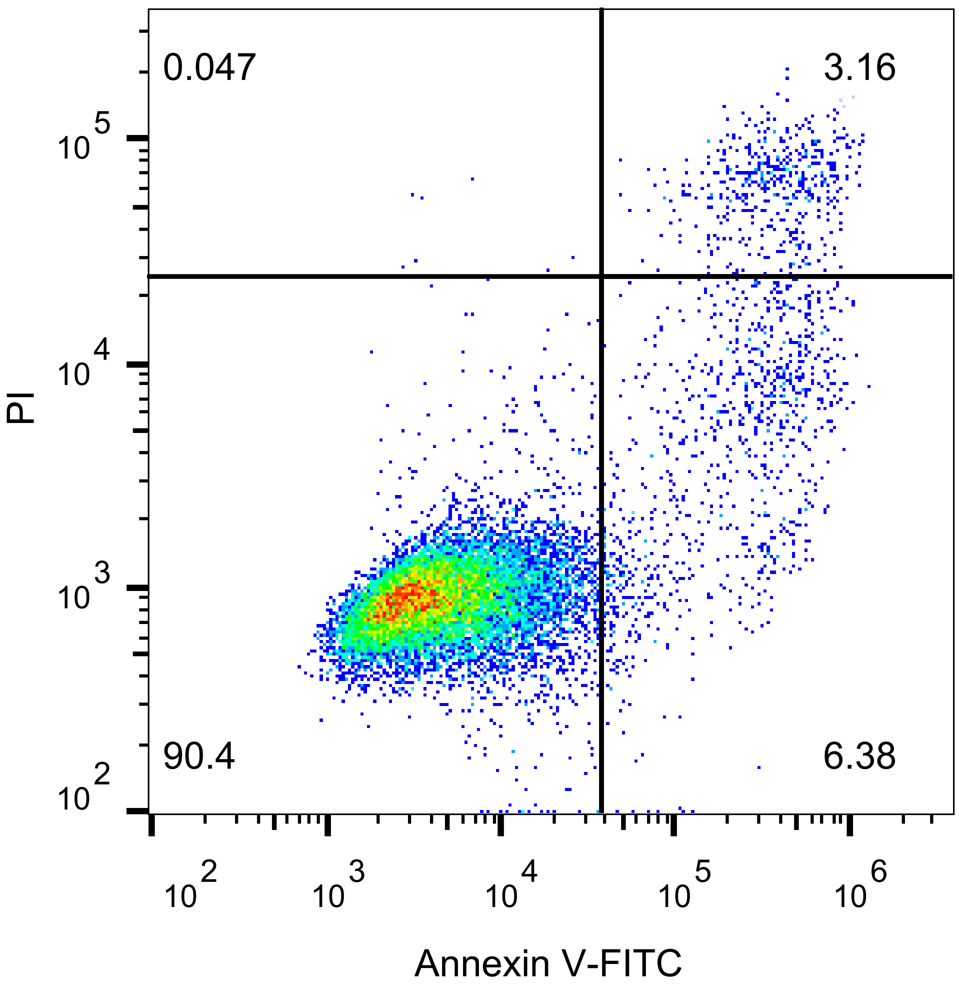

HF-3.fcs

FSC-A, FSC-Width subset

14901

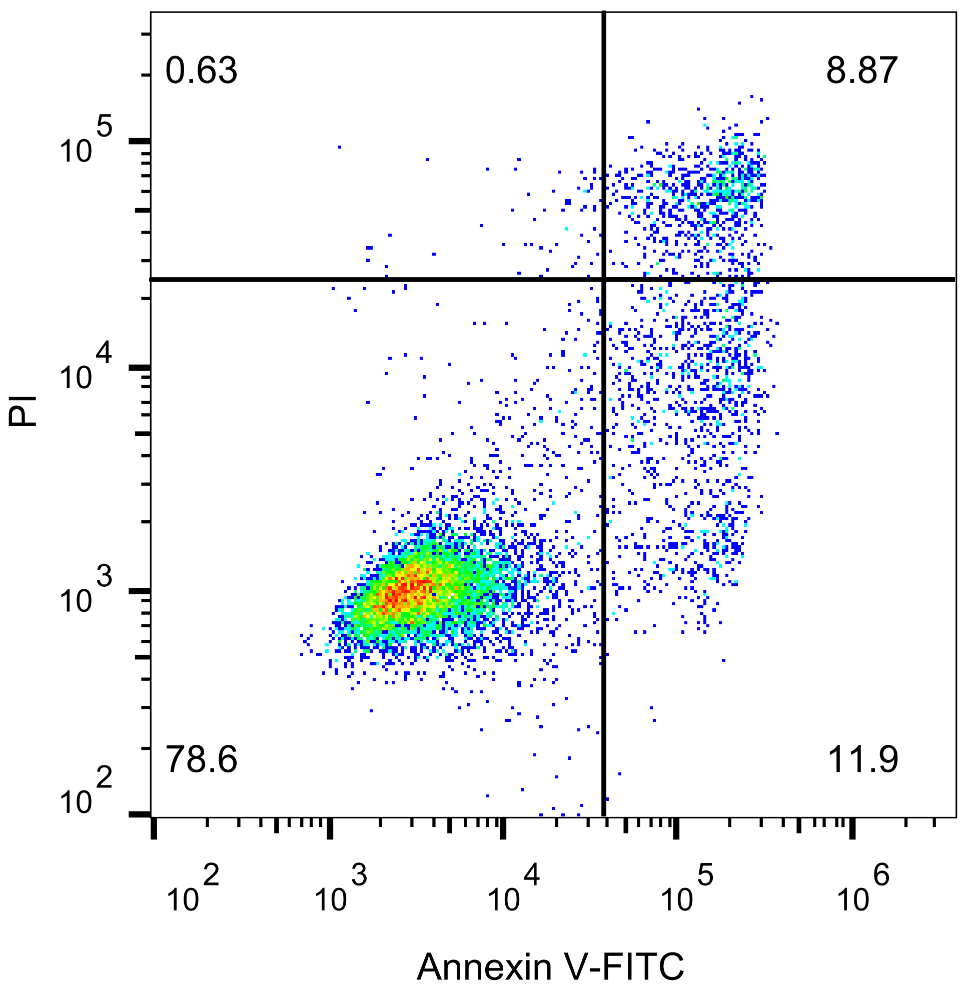

HF-20-1.fcs

FSC-A, FSC-Width subset

10329

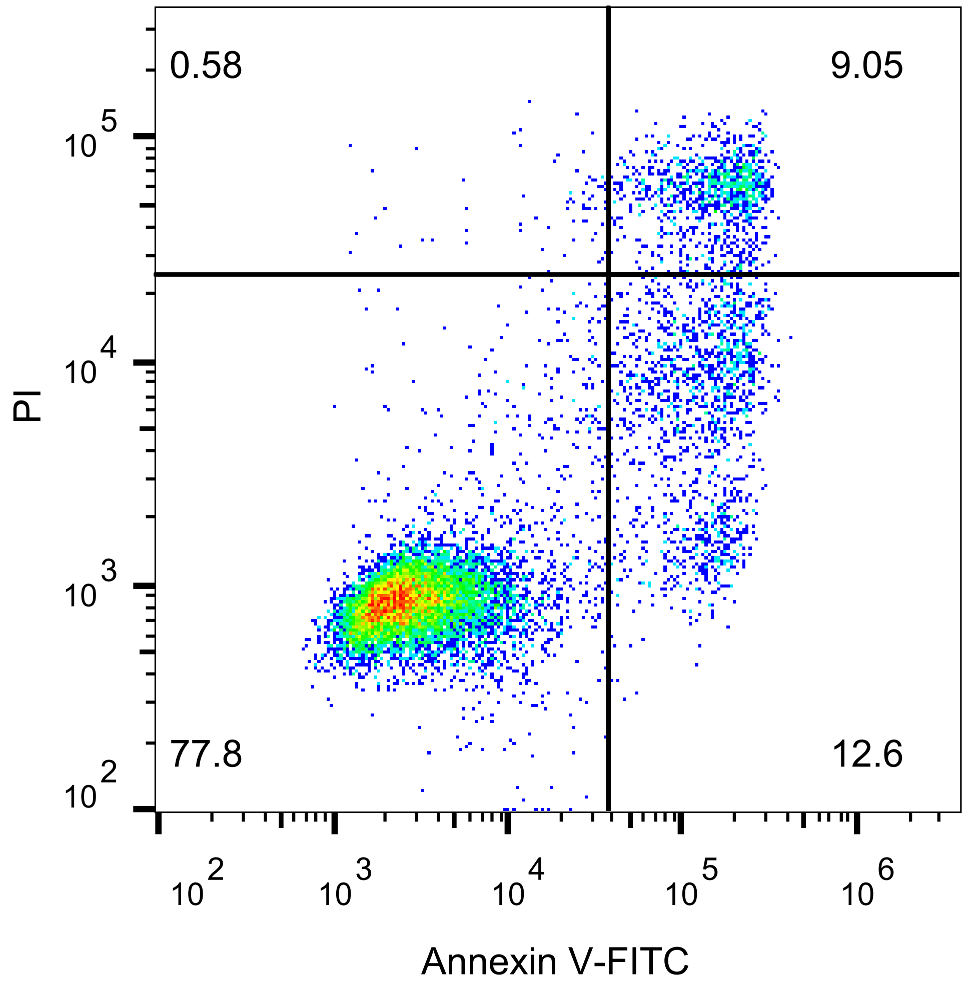

HF-20-2.fcs

FSC-A, FSC-Width subset

12126

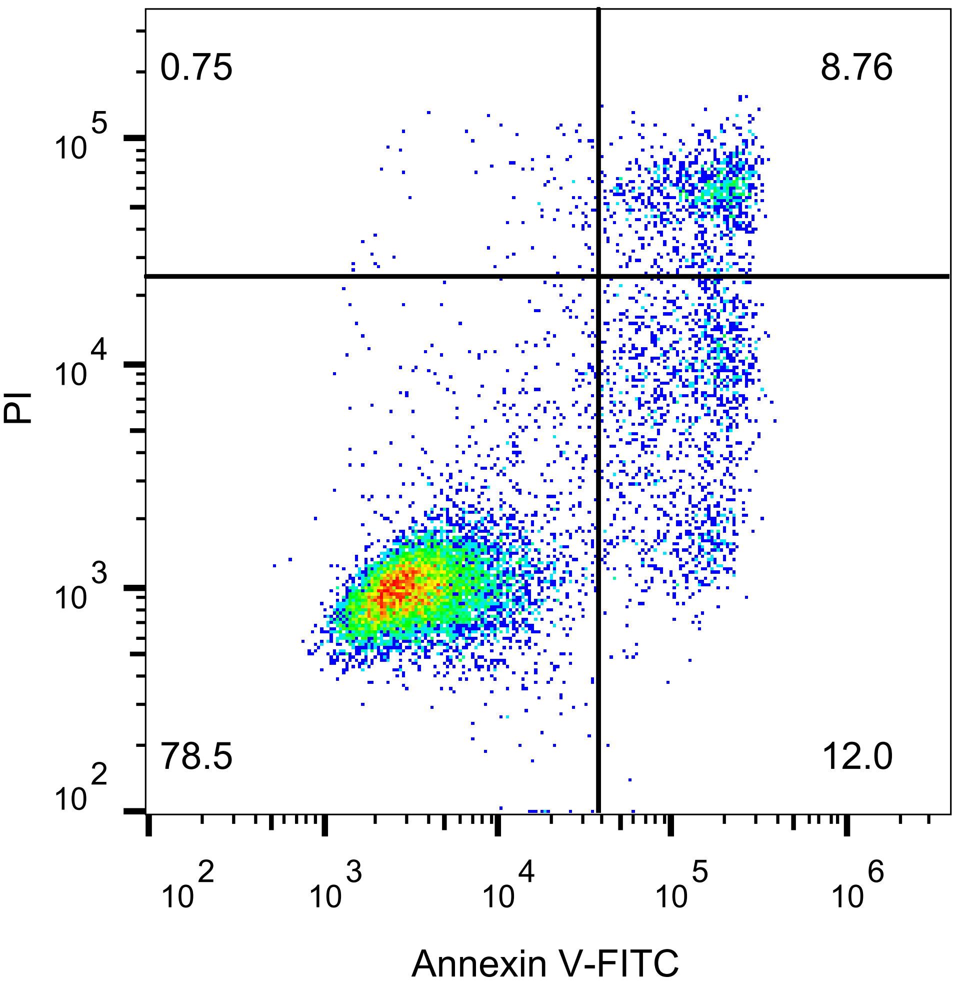

HF-20-3.fcs

FSC-A, FSC-Width subset

11728

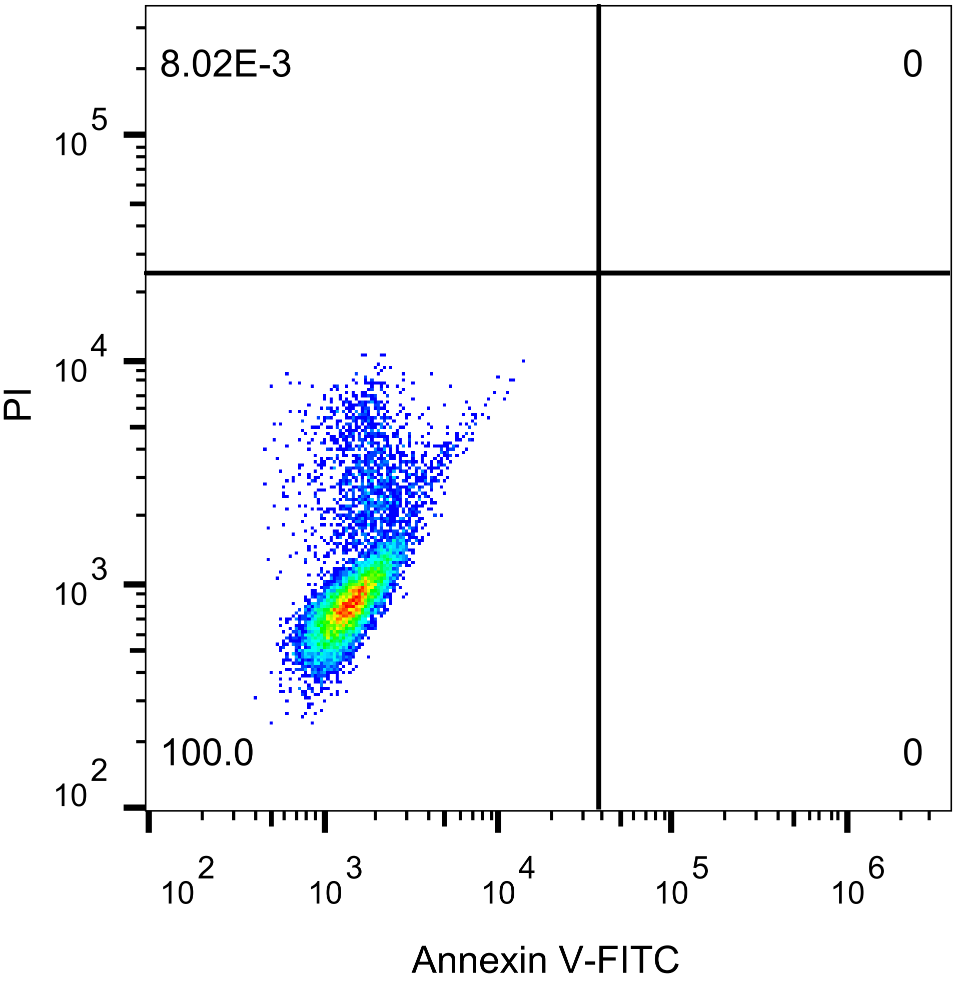

men.fcs

FSC-A, FSC-Width subset

12476
